# Mcl-1 is a key regulator of the ovarian reserve

**DOI:** 10.1038/cddis.2015.95

**Published:** 2015-05-07

**Authors:** S Omari, M Waters, T Naranian, K Kim, A L Perumalsamy, M Chi, E Greenblatt, K H Moley, J T Opferman, A Jurisicova

**Affiliations:** 1 Lunenfeld Tanenbaum Research Institute, Mount Sinai Hospital, 25 Orde Street, Toronto, Ontario M5T 3H7, Canada; 2 Department of Physiology, University of Toronto, 1 King's College Circle, Toronto, Ontario M5S 1A8, Canada; 3 Department of Obstetrics and Gynecology, Washington University in St. Louis, 660S Euclid Avenue, St. Louis, MO 63110, USA; 4 Centre for Fertility and Reproductive Health, Mount Sinai Hospital, 250 Dundas Street, Toronto, Ontario M5T 2Z5, Canada; 5 Department of Obstetrics and Gynecology, University of Toronto, 92 College Street, Toronto, Ontario M5G 1L4, Canada; 6 Department of Biochemistry, St. Jude Children's Research Hospital, MS 340, Room D4063D, 262 Danny Thomas Place, Memphis, TN 38105, USA

## Abstract

A majority of ovarian follicles are lost to natural death, but the disruption of factors involved in maintenance of the oocyte pool results in a further untimely follicular depletion known as premature ovarian failure. The anti-apoptotic B-cell lymphoma 2 (Bcl-2) family member myeloid cell leukemia-1 (MCL-1) has a pro-survival role in various cell types; however, its contribution to oocyte survival is unconfirmed. We present a phenotypic characterization of oocytes deficient in *Mcl-1*, and establish its role in maintenance of the primordial follicle (PMF) pool, growing oocyte survival and oocyte quality. *Mcl-1* depletion resulted in the premature exhaustion of the ovarian reserve, characterized by early PMF loss because of activation of apoptosis. The increasingly diminished surviving cohort of growing oocytes displayed elevated markers of autophagy and mitochondrial dysfunction. *Mcl-1-*deficient ovulated oocytes demonstrated an increased susceptibility to cellular fragmentation with activation of the apoptotic cascade. Concomitant deletion of the pro-apoptotic Bcl-2 member Bcl-2-associated X protein (*Bax*) rescued the PMF phenotype and ovulated oocyte death, but did not prevent the mitochondrial dysfunction associated with *Mcl-1* deficiency and could not rescue long-term breeding performance. We thus recognize MCL-1 as the essential survival factor required for conservation of the postnatal PMF pool, growing follicle survival and effective oocyte mitochondrial function.

Estimates of the human primordial follicle (PMF) reservoir, the size of which dictates the extent of the ovarian reserve, indicates the presence of at least half a million oocytes per ovary at birth.^1, 2^ The essential decision that PMFs face is either long-term arrest with a possibility of recruitment toward the growing pool, or death. Even upon recruitment to the growing pool, intricately orchestrated crosstalk of survival signals between ovarian somatic cells and oocytes facilitate the ovulation of a single oocyte in human in each cycle. Hence, the default fate for millions of ovarian germ cells is death, as only a small fraction survive till ovulation.^3^ Insufficient endowment during fetal development or excessive oocyte loss during postnatal life further limits the ovarian reserve and can result in an untimely exhaustion of the follicle pool leading to premature ovarian failure (POF); a syndrome that affects around 1% of all women, with a higher prevalence (up to 30%) in families with heritable traits of this condition.^4, 5^ Mechanisms responsible for maintenance of the follicular reserve are poorly understood, however, biological assessments and mathematical modeling reveal that progressive loss of follicles with age is non-linear and accelerates, especially after 38 years.^6, 7^ With a declining ovarian reserve, poor oocyte quality is an additional factor that contributes to the reduced fertility associated with increased maternal age. Oocytes and resulting embryos of older mothers have increased rates of aneuploidies likely due to defects in chromosomal cohesion and meiotic spindle stability, decreased DNA repair capacity, altered gene expression, impaired mitochondrial function and elevated cellular redox, all contributing to increased rates of cell death.^8, 9, 10^

The marked decline of oocyte number in mammalian ovaries has been attributed to oocyte loss via stage-specific modes of death. As yet, perinatal PMF loss in mice most frequently engages apoptotic cell death,^11, 12^ whereas within the postnatal ovary, oocytes in growing follicles undergo atresia, a less 'molecularly' defined death, carrying hallmarks of both apoptosis and autophagy.^13, 14, 15^ It is thus surprising that no member of the anti-apoptotic B-cell lymphoma 2 (Bcl-2) family has been identified with a definitive role in governing oocyte survival and the maintenance of the ovarian reserve. *Bcl-2l2*/Bcl-w and *Bcl-2-l10*/Diva deficiency had no apparent impact on the ovarian reserve, and although ablation of *Bcl-2* led to a loss of one-third of the adult PMF pool, the growing follicle pool was not significantly impacted and these animals did not undergo POF.^16, 17, 18, 19^ Conditional Bcl-x *(Bcl-2l1)* inactivation led to increased primordial germ cell apoptosis in the embryo,^20^ but postnatal inactivation of *Bcl-x* in oocytes did not compromise the ovarian reserve in young females.^21^ *Bcl2a1a*/*Bfl-1*/*A1* was low to undetectable in fully grown germinal vesicle (GV) or ovulated murine oocytes,^22^ however, the impact of *Bfl-1* deficiency on the ovarian reserve has not yet been analyzed to the best of our knowledge. Consequently, either various anti-apoptotic Bcl-2 members have overlapping roles in governing postnatal oocyte survival and maintenance of the adult ovarian reserve in mice, or the anti-apoptotic Bcl-2 member that regulates this decision has yet to be identified.

## Results

### Myeloid cell leukemia-1 (MCL-1) in oocytes is reduced in association with aging and activation of follicle atresia

**Figure 1:**
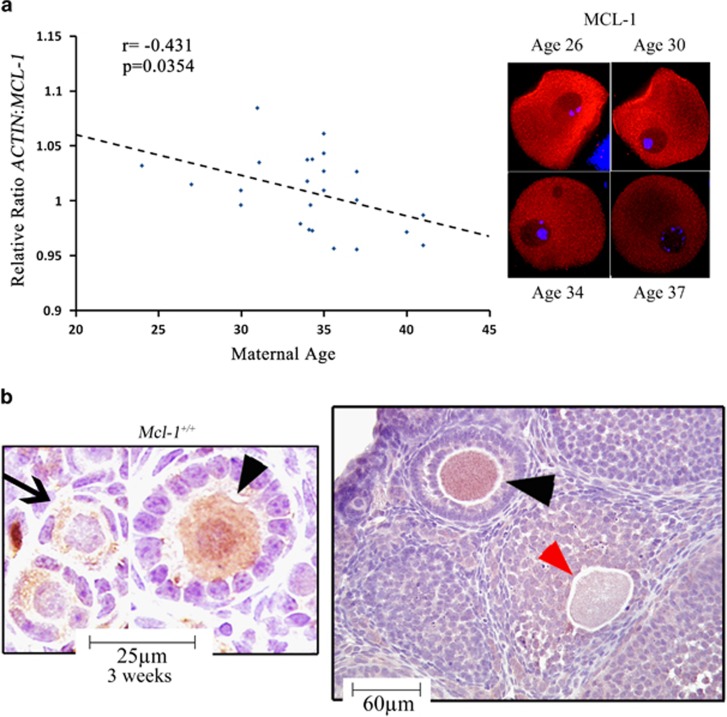
Patterns of MCL-1 expression in human and murine oocytes. (**a**) Age-associated changes in *MCL-*1 transcript (left) and MCL-1 protein immunoreactivity (right) in human GV oocytes from different patients. Pearson analysis revealed a negative correlation between *MCL-1* transcript level and maternal age (*n*=24; *r*=−0.431; *P*=0.035). (**b**) Immunohistochemical staining of MCL-1 (brown) in 3-week ovarian sections of wild-type mouse ovaries, counterstained with hematoxylin (blue). PMF oocytes (arrows), PF (PF) and PA follicles (arrowheads), and oocytes undergoing early stages of atresia (red arrowhead). At least three ovaries were stained per age group with identical expression patterns

With the majority (99%) of all oocytes undergoing some form of programmed cell death (PCD),^3, 13, 14, 15, 23^ and only a few Bcl-2 family members identified with roles in regulation of germ cell fate,^16, 17, 18, 19, 20, 21^ we set to investigate which members of the anti-apoptotic Bcl-2 gene family decline with maternal age in human GV stage oocytes. A reduction in age-dependent oocyte quality has been previously associated with activation of cell death or molecular pathways that regulate this fate.^8, 10^ From the analyzed targets (*BCL-2, BCL-2L1, BCL-2L10*, *BCL-2L11* and *MCL-1*), only the *MCL-1* transcript significantly declined in an age-associated manner, with a decrease in MCL-1 immunoreactivity in human GV oocytes (Figure 1a). Changes in *MCL-1* transcript associated with differences in clinical diagnoses, stimulation protocol or resultant outcome proved nonsignificant (Supplementary Table S2). MCL-1 has been shown to be expressed by fetal human and neonatal mouse oocytes,^16, 24^ and abundant transcript levels were detected in ovulated human oocytes.^25^ We assessed the expression of MCL-1 in murine ovaries and before pubertal onset cytoplasmic immunoreactivity of MCL-1 in oocytes increased with activation of follicle growth (from PMF to primary follicle (PF)), remained robust in the fully grown oocytes of preantral (PA) follicles and virtually disappeared from oocytes undergoing atresia (Figure 1b). This pattern indicated that MCL-1 could be an essential regulator of oocyte survival.

### Oocyte-specific *Mcl-1* deficiency reduces ovarian follicle pool, ovulation and fertility

To determine the functional requirement for *Mcl-1* in oogenesis, we created a mouse model with oocyte-specific excision of *Mcl-1* using zona pellucida 3 (Zp3)-Cre. We confirmed spatial and temporal activity of the Cre transgene by introduction into two reporter lines, tdTomato and lacZ/alkaline phosphatase reporter (Z/AP). Initial activation of Cre excision in oocytes was observed as early as embryonic day 17.5, with a large proportion of oocytes showing excision by postnatal day 3 (PN3; Supplementary Figure S1A). The selectivity of excision was also confirmed in the adult Z/AP reporter line, with excision in virtually all growing oocytes (Supplementary Figure S1B). We next combined the Zp3-Cre allele with floxed *Mcl-1* (*Mcl-1*^*f/f*^). Effective excision was confirmed by lack of MCL-1 immunoreactivity in oocytes of *Mcl-1*^*f/−*^: Zp3-Cre (Mcl-1 conditional knockout (*Mcl-1*cKO)) females (Supplementary Figure S1C).

**Figure 2:**
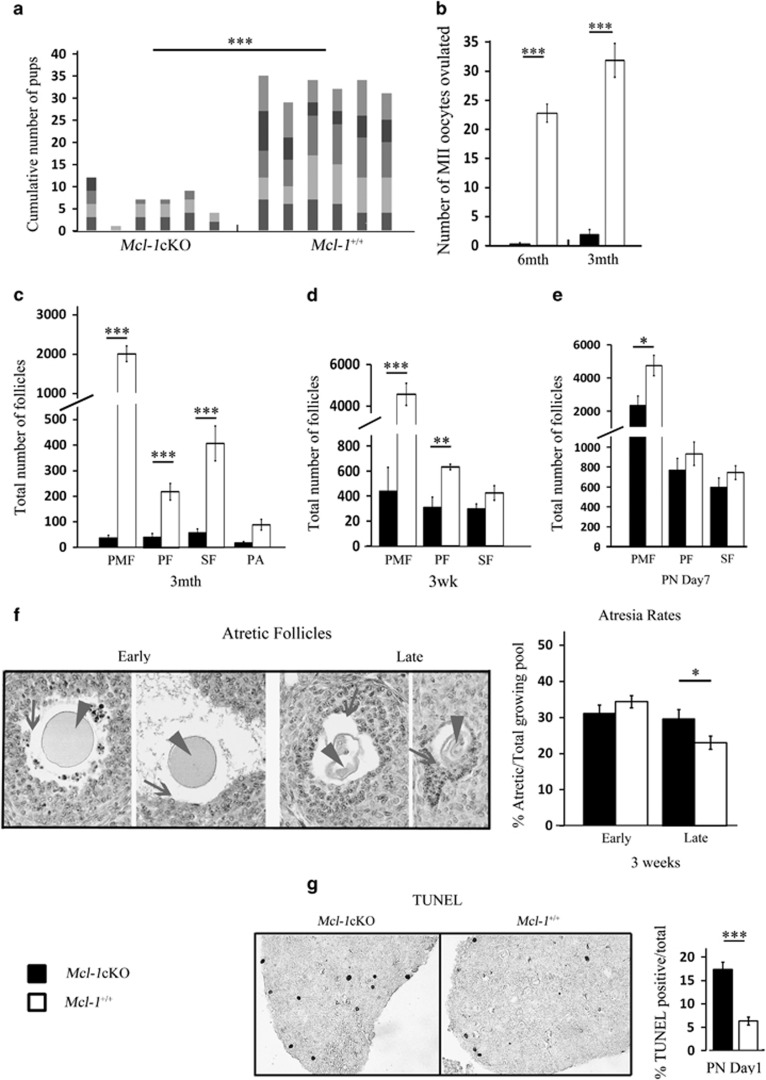
Breeding performance, ovulation rates and histomorphometric analyses of ovaries. (**a**) Cumulative pup number from *Mcl-1*cKO (*n*=6) and *Mcl-1*^*+/+*^(*n*=6) females bred with wild-type males at 4 weeks for 6-month trial. Columns represent individual females with each varied color segment indicative of individual litters. (**b**) Ovulation rates from primed 6-month *Mcl-1*cKO (*n*=12), compared with *Mcl-1*^*+/+*^ (*n*=18) control females and 3-month *Mcl-1*cKO (*n*=20), compared with *Mcl-1*^*+/+*^ (*n*=12) females. Values represent average oocytes ovulated per female±S.E.M. (**c**) Histomorphometric analyses of PMF, PF, SF and PA follicles in 3-month *Mcl-1*cKO (*n*=5), compared with *Mcl-1*^*+/+*^ (*n*=3) females; (**d**) in 3-week ovaries of *Mcl-1*cKO (*n*=5) to *Mcl-1*^*+/+*^ (*n*=4) females; (**e**) and in day 7 (PN7) *Mcl-1*cKO (*n*=4), compared with *Mcl-1*^*+/+*^ (*n*=4) females. Values in **c, d** and **e** represent average number of follicles/ovary±S.E.M. (**f**) Representative stages of follicle atresia (left) in ovarian sections stained with methyl green, display granulosa cell death/detachment (arrows) preceding oocyte shrinkage (arrowhead). Both early and late-stage follicle atresia rates of *Mcl-1*cKO (*n*=4), compared with *Mcl-1*^*+/+*^ (*n*=5) ovaries. Atretic follicles were taken as a proportion of the total post-secondary growing follicles and separated into advanced stage (late) or early/moderate stage follicle atresia. (**g**) Death rates based on TUNEL stain of PN1 ovaries of *Mcl-1*cKO (*n*=4) compared with *Mcl-1*^*+/+*^ (*n*=3) females counterstained with methyl green. Values represent average percentage of TUNEL positive/total PMF per section±S.E.M. Significance denoted by number of stars (*P<0.05, **P<0.01, ***P<0.001). Genotypes in legend apply to all graphs

To determine the impact of *Mcl-1* oocyte-specific deletion on fertility, *Mcl-1*cKO and control females were bred to wild-type males of proven fertility for a period of 6 months. The cumulative breeding performance of *Mcl-1*cKO dams was markedly reduced with an average of two litters during this period, and each with less than half of the pup number obtained from control females (Figure 2a, Supplementary Figure S2A). In addition, *Mcl-1*cKO females did not deliver any live litters beyond 4 months of age, whereas all control females of the various genotypes were able to breed beyond 1 year of age (Supplementary Figure S2A). *Mcl-1* excision was confirmed in all genotyped *Mcl-1*cKO offspring (*n*=31). To establish whether the reduction in breeding performance was due to an overall reduction in the ovulatory capacity, females were primed with external gonadotropins and their ovulatory response quantitated. We used females of various age groups (at 6 and 3 months, as well as 3 weeks) as these represented mature females, at a young reproductive age and at pubertal onset, respectively. In *Mcl-1*cKO females, very poor ovulatory capacity was observed at 3 months and ovulation was virtually absent by 6 months (Figure 2b, Supplementary Figure S2B). Histological examination revealed a severe depletion in PMF, PF and secondary follicles (SF) and a drastic reduction in ovary size already evident at 3 months of age (Figure 2c, Supplementary Figure S2D). Furthermore, a significant reduction of PMFs and PFs was also observed in heterozygote females (Supplementary Figure S2C). Interestingly, at the onset of puberty (3 weeks), females of all genotypes ovulated comparable numbers of oocytes (Supplementary Figure S2E), yet histomorphometric analyses revealed a sharp reduction in PMFs and a significant decrease in growing follicle number (Figure 2d). The loss of growing oocytes may be partially due to an increased rate of atresia, as a significantly higher proportion of late-stage atretic follicles was observed (Figure 2f).

The loss of the resting PMF pool was the most marked outcome we have observed. At PN7, only about half of the PMFs were present with no change in the PF or SF population (Figure 2e). This, combined with terminal deoxynucleotidyl transferase dUTP nick end labeling (TUNEL) analyses of ovaries at PN1 revealing that *Mcl-*1 deficiency resulted in a doubling of the number of apoptotic (TUNEL positive) oocytes compared with controls (Figure 2g, Supplementary Figure S2F), led us to conclude that *Mcl-1* is required for postnatal maintenance of the ovarian reserve.

### *Mcl-1* deficiency leads to elevated activation of autophagy in antral follicle population

Based on our observations that *Mcl-1* ablation resulted in mildly elevated atresia rates, and that MCL-1 expression was absent from oocytes undergoing atresia, we next assessed markers of PCD in the growing follicle pool. As PMF oocytes of *Mcl-1*cKO ovaries die via an apparent apoptotic cell death, we set to investigate whether antral GV oocytes also activate this death pathway. Relative to controls, *Mcl-1*cKO oocytes exhibited an increase in Bcl-2-associated X protein (BAX)-NT, indicative of a conformational change in the N-terminus of BAX and potentiating oligomerization; however, downstream hallmarks of mitochondrial engagement (e.g., cytochrome C or apoptosis-inducing factor (AIF) release) showed no significant change, with no increase in pan-caspase activity (Supplementary Figures S3A–D). This data effectively demonstrates that despite BAX activation, there is no subsequent instigation of the apoptotic cascade in antral *Mcl-1*cKO GV oocytes.

**Figure 3:**
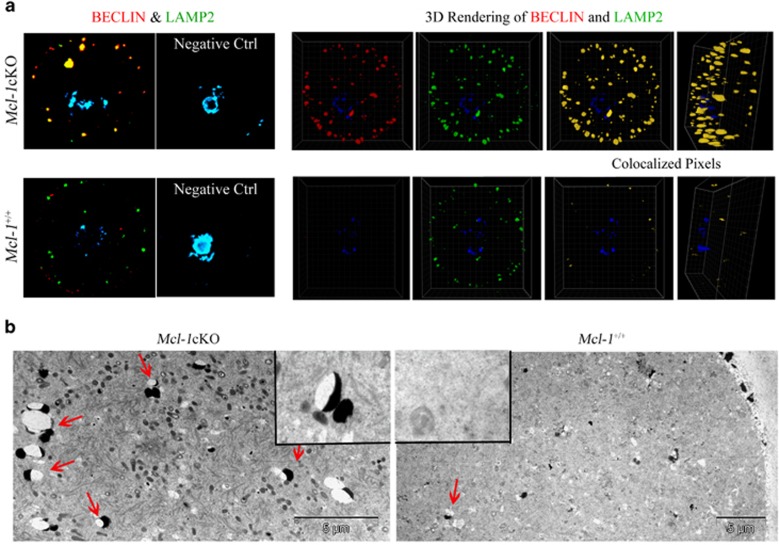
Markers of autophagy in GV oocytes. (**a**) Confocal image of Beclin-1 (red) and LAMP-2 (green) foci in isolated GV oocytes from 3-week ovaries, counterstained with DAPI (blue). To determine colocalization, 3D rendering of all layers was performed on *n*=19 *Mcl-1*cKO and *n*=13 *Mcl-1*^*+/+*^GV oocytes with colocalized (yellow) pixels displayed. Coefficients of colocalization for *Mcl-1*cKO oocytes compared with *Mcl-1*^*+/+*^(Pearsons (PCC)=0.51±0.05 to 0.17±0.07, respectively; *P*<0.001) (Manders A (MCC-A)=0.098±0.017 to 0.058±0.01, respectively; *P*<0.05) (Manders B (MCC-B)=0.079±0.014 to 0.032±0.005, respectively; *P*<0.05) (**b**) Transmission electron microscopy (TEM) of *Mcl-1*cKO and wild-type control GV oocytes with lysosome-associated vesicles (red arrows) indicated

As follicle atresia exhibits hallmarks of both autophagic (PCD type 2) and apoptotic (PCD type 1) cell death^13^ and Mcl-1 has been previously linked to inhibition of both pathways,^26, 27, 28^ we turned to the assessment of markers of autophagic activation in GV oocytes. Induction or phosphorylation of Beclin-1/BECN-1 has been shown to initiate autophagosome formation,^29^ and MCL-1 binding and inhibition of Beclin-1 prevented autophagy.^27^ *Mcl-1*cKO oocytes displayed an increase in size and number of Beclin-1 foci, indicating an increase in Beclin-1-associated vesicle formation (Figure 3a,Supplementary Figure S3F). In addition, microtubule-associated protein 1 light chain (MAP1LC3A), involved in autophagosome membrane elongation,^30^ was elevated in *Mcl-1*cKO GV oocytes (Supplementary Figure S3E). We next examined markers of late autolysosomes. Lysosome-associated membrane proteins 1 and 2 (LAMP-1, LAMP-2) are an essential part of autophagosome maturation, autolysosome formation and lysosomal fusion.^31^ *Mcl-1*cKO GV oocytes showed an increased number of foci positive for both Beclin-1 and LAMP-2 (Figure 3a). Colocalization analyses performed on 3D rendered oocyte sections confirmed a significant increase in Beclin-1/LAMP-2 colocalization in *Mcl-1*cKO GV oocytes. These colocalization analyses also demonstrated that oocytes possessing just one *Mcl-1* allele (*Mcl-1*^*f/+*^: Zp3-Cre), display an intermediary phenotype (Supplementary Figure S3F). The amplified abundance of autolysosome structures in *Mcl-1*cKO oocytes was further validated by transmission electron microscopy (Figure 3b). The elevation in number of Beclin-1-positive foci, LC-3 immunoreactivity and Beclin-1/LAMP-2 colocalized structures, indicates an augmentation of autophagosome and autolysosome formation, triggered by *Mcl-1* deficiency.

### Ovulated *Mcl-1*-deficient oocytes display mitochondrial dysfunction and chromosomal abnormalities

**Figure 4:**
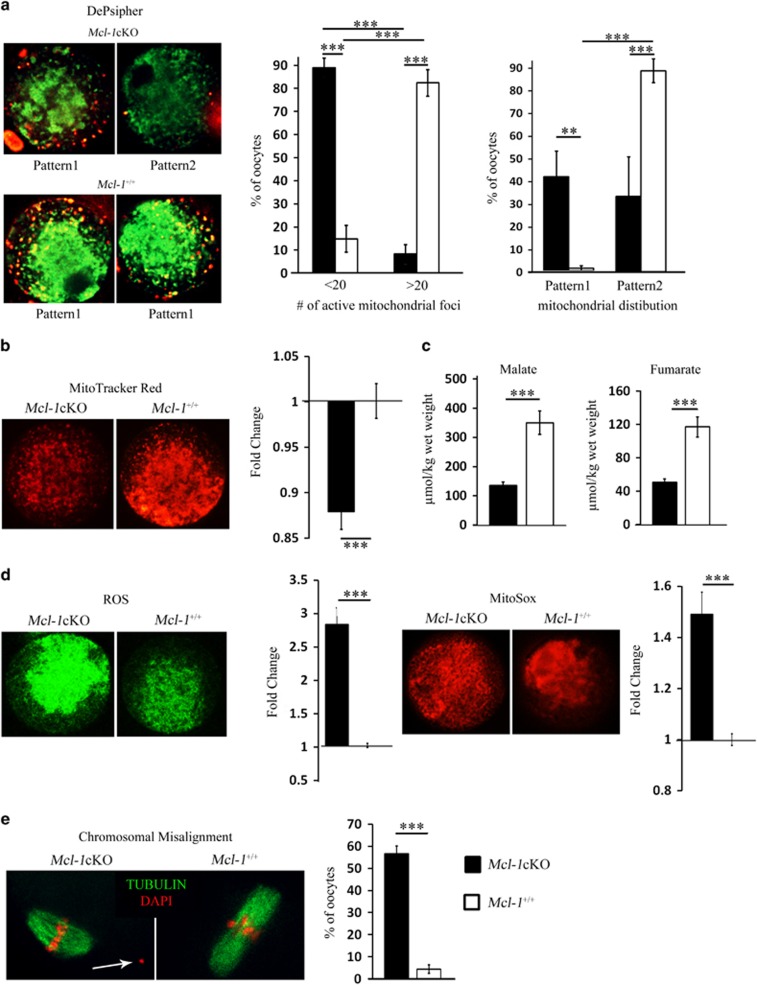
Markers of mitochondrial function and chromosomal alignment. (**a**) *Mcl-1*cKO (*n*=57) and *Mcl-1*^*+/+*^ (*n*=85) MII oocytes were stained with a potential dependent mitochondrial dye (DePsipher). Values in graph (left) represent a proportion (%) of oocytes with few (<20) or numerous (>20) polarized (red) mitochondrial foci±S.E.M. per *Mcl-1*cKO or *Mcl-1*^*+/+*^ oocytes. Values in graph (right) represent the proportion (%) of oocytes with mitochondrial distribution separated into pattern 1 or pattern 2±S.E.M. (**b**) Mean intensity of *Mcl-1*cKO (*n*=68) and *Mcl-1*^*+/+*^ (*n*=52) MII oocytes stained with MitoTracker Red. Values represent average fold change±S.E.M. of relative fluorescence units (RFUs) per oocyte, normalized to wild type. (**c**) *Mcl-1*cKO (*n*=15) and *Mcl-1*^*+/+*^ (*n*=15) oocytes were assayed for levels of TCA cycle substrates malate and fumarate. Values represent average metabolite level (*μ*mol) per oocyte wet weight (kg)±S.E.M. (**d**) MII oocytes were stained with DCFDA (green) (*Mcl-1*cKO (*n*=20) and *Mcl-1*^*+/+*^ (*n*=17)) or with MitoSox (red) (*Mcl-1*cKO (*n*=20) and *Mcl-1*^*+/+*^ (*n*=24)). Values represent average fold change±S.E.M. of RFUs per oocyte, normalized to wild type. (**e**) MII oocytes were stained with DAPI (red) and anti-tubulin (green) to visualize chromatin and spindle; chromosomal misalignments (white arrow) from *Mcl-1*cKO (*n*=84) and *Mcl-1*^*+/+*^ (*n*=89) oocytes were quantitated. Values represent percentage of oocytes with misaligned chromosomes/total oocyte pool. (*P<0.05, **P<0.01, ***P<0.001). Genotypes in legend apply to all graphs

Despite the marked loss of the ovarian pool at a young age, *Mcl-1*cKO females do ovulate and breed, albeit at reduced efficiencies. As oocyte quality and reproductive competence have been suggested to rely on effective mitochondrial metabolism,^32, 33, 34^ and Mcl-1 has been implicated in regulation of mitochondrial function in somatic cells,^28, 35, 36^ we assessed these parameters in *Mcl-1*cKO ovulated oocytes. Using a mitochondrial membrane potential sensitive dye, we observed two distinct patterns of DePsipher fluorescence (Figure 4a). Control oocytes displayed a strong centrally localized inactive mitochondrial pool (green) with enrichment of polarized mitochondria (red) in the cortical region (pattern 1). Conversely, many *Mcl-1*cKO oocytes exhibited a weak diffuse distribution of inactive mitochondria (green) and extremely few peripherally active polarized mitochondria (red) (pattern 2). Moreover, the number of peripheral polarized mitochondrial foci was significantly decreased in *Mcl-1*cKO oocytes compared with controls (Figure 4a,Supplementary Figure S4A). A decline in the respiring mitochondrial pool, evidenced by decreased MitoTracker Red intensity was also observed in *Mcl-1*cKO metaphase II (MII) oocytes (Figure 4b,Supplementary Figure S4C). This decrease was significant despite a marked elevation in the total mitochondrial pool demonstrated by increased MitoTracker Green (Supplementary Figure S4B), a dye that binds mitochondrial membranes irrespective of their activity. In measuring mitochondrial output, *Mcl-1*cKO MII oocytes presented with a sharp reduction in levels of the TCA cycle substrates fumarate and malate (Figure 4c), but showed no apparent difference in total ATP level or citrate (Supplementary Figure S4D), when compared with control, implying no change in available energy. In addition, total reactive oxygen species (ROS) and mitochondrial-derived superoxides were markedly increased (Figure 4d, Supplementary Figures S4E and 4F). High ROS levels can be indicative of defective antioxidant machinery or an inhibition/block in the electron transport chain,^37, 38^ and elevated ROS and impaired oxidative phosphorylation have been linked with defective development and meiotic spindle defects.^33, 39, 40, 41^ Microtubule staining of the meiotic spindle apparatus revealed increased chromosomal misalignments in *Mcl-1*cKO oocytes compared with controls with misaligned chromosome(s) not attached to spindle (Figure 4e, Supplementary Figure S4G).

### *Mcl-1*-deficient ovulated oocyte pool displays elevated markers of apoptosis and increased fragmentation rates

**Figure 5:**
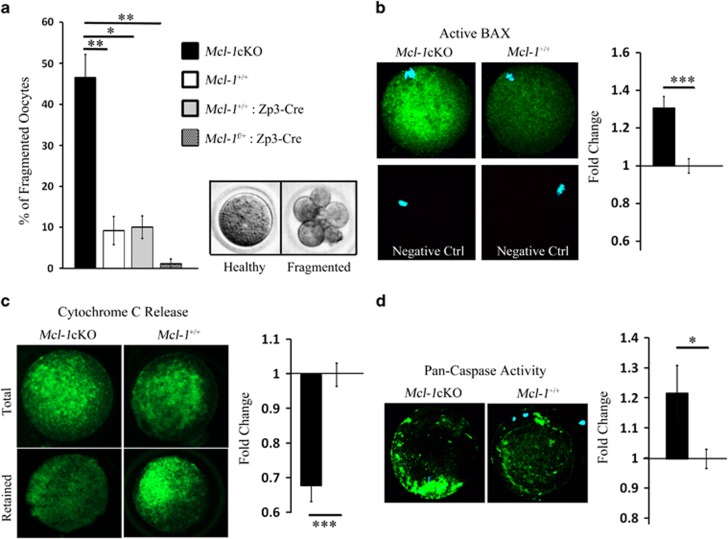
Markers of apoptosis in ovulated oocytes. (**a**) Oocytes obtained from *Mcl-1*cKO (*n*=6), *Mcl-1*^*+/+*^ (*n*=9), *Mcl-1*^*+/+*^: Zp3-Cre (*n*=8), and *Mcl-1*^*f/+*^: Zp3-Cre (*n*=4) females (*n*=104, *n*=180, *n*=241 and *n*=69 oocytes, respectively) were scored 24 h after *in vitro* culture for percentage of fragmented oocytes±S.E.M. per female/genotype. (**b**) *Mcl-1*cKO (*n*=12) and *Mcl-1*^*+/+*^ (*n*=13) MII oocytes were stained with anti-BAX-NT (green), counterstained with DAPI (blue). Mean intensity was quantitated as fold change of RFUs per oocyte±S.E.M. normalized to average *Mcl-1*^*+/+*^ value. (**c**) *Mcl-1*cKO (*n*=19) and *Mcl-1*^*+/+*^ (*n*=33) MII oocytes were stained for total, and *Mcl-1*cKO (*n*=13) and *Mcl-1*^*+/+*^ (*n*=31) oocytes, were stained for retained Cytochrome c (green). Values represent the average fold change of RFUs per oocyte±S.E.M. of retained/total cytochrome c, normalized to average *Mcl-1*^*+/+*^ value. (**d**) *Mcl-1*cKO (*n*=16) and *Mcl-1*^*+/+*^ (*n*=13) MII oocytes were stained for pan-caspase activity (green). Values indicate average fold change of RFUs per oocyte±S.E.M. normalized to average *Mcl-1*^*+/+*^ value (*P<0.05, **P<0.01, ***P<0.001). Genotypes in legend apply to all graphs

Defective mitochondrial bioenergetics are expected to impair oocyte survival, particularly after ovulation, when cumulus cells stop directly providing oocytes with nutrients.^42^ Thus, we decided to investigate whether the viability of ovulated oocytes was compromised because of *Mcl-1* deficiency. *In vitro* culture revealed an inability of *Mcl-1*cKO MII oocytes to sustain meiotic arrest over 24 h and an increased propensity to undergo cellular fragmentation (Figure 5a). As cellular fragmentation of oocytes is thought to occur via apoptosis,^43^ we explored whether hallmarks of activation of the apoptotic pathway could be detected. Indeed, ovulated oocytes displayed an increase in BAX-NT (Figure 5b, Supplementary Figure S5A) with no change in total BAX levels (Supplementary Figure S5A), accompanied by an increase in mitochondrial cytochrome C release (Figure 5c, Supplementary Figure S5B) and caspase activity (Figure 5d, Supplementary Figure S5C). Thus, *Mcl-1* deficiency in ovulated MII oocytes leads to an activation of the apoptotic cascade, and an increased proclivity for these compromised oocytes to fragment.

### Concurrent deletion of *Bax* rescues ovarian follicle loss and increased fragmentation rate but does not impact mitochondrial dysfunction or chromosome abnormalities

**Figure 6:**
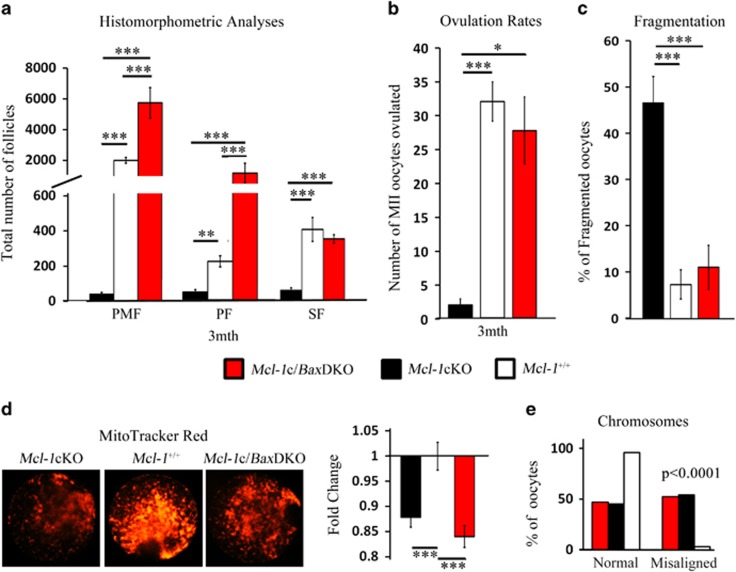
Rescue of *Mcl-1*-deficient follicle loss by concurrent Bax ablation. (**a**) Histomorphometric analyses of 3-month ovaries in *Mcl-1*cKO (*n*=5), *Mcl-1*^*+/+*^(*n*=3) and *Mcl-1*c/*Bax*DKO (*n*=3) females. Values represent average follicle number in ovary per genotype±S.E.M. (**b**) Ovulation rates of *Mcl-1*cKO (*n*=20), *Mcl-1*^*+/+*^(*n*=12) and *Mcl-1*c/*Bax*DKO (*n*=3) 3-month females. Values represent average number of ovulated oocytes±S.E.M. (**c**) MII oocytes from *Mcl-1*cKO (*n*=6), *Mcl-1*^*+/+*^(*n*=10) and *Mcl-1*c/*Bax*DKO (*n*=3) females (*n*=104, *n*=180, *n*=48 oocytes, respectively) cultured for 24 h and values represent the average percentage of fragmented oocytes per female±S.E.M. (**d**) *Mcl-1*cKO (*n*=50), *Mcl-1*^*+/+*^(*n*=33), and *Mcl-1*c/*Bax*DKO (*n*=49) MII oocytes were stained with MitoTracker Red. Values represent fold change of average RFUs per oocyte±S.E.M. normalized to average *Mcl-1*^*+/+*^ value. (**e**) Chromosomal misalignments in *Mcl-1*cKO (*n*=70), *Mcl-1*^*+/+*^ (*n*=89) and *Mcl-1*c/*Bax*DKO (*n*=19) oocytes were quantitated (from *n*=4, *n*=5 and *n*=2 animals, respectively). Values represent percentage of oocytes with misaligned or normal chromosomes/total oocyte pool. *Mcl-1*c/*Bax*DKO data were all graphed against *Mcl-1*cKO and *Mcl-1*^*+/+*^data from Figures 2,4 and 5. (*P<0.05, **P<0.01, ***P<0.001). Genotypes in legend apply to all graphs

Deletion of the pro-apoptotic Bcl-2 family member *Bax* rescues the primordial germ cell loss induced by ablation of the anti-apoptotic *Bcl-x*.^20^ By itself, Bax deficiency has been linked to increased primordial germ cell survival,^44^ resulting in an increase in follicular endowment^45^ and extended ovarian function to advanced chronological age.^46^ In order to evaluate whether Bax deficiency would also rescue loss of the ovarian reserve in *Mcl-1*cKO females, we crossed a *Bax*-deficient line to our *Mcl-1*cKO mouse line. Ovaries from 3 month *Bax*^*−/−*^(Bax knockout (*Bax*KO)), *Mcl-1*^*f/−*^: *Bax*^*−/−*^: Zp3-Cre (double knockout with conditional Mcl-1 and total Bax deletion (*Mcl-1*c*/Bax*DKO)), *Mcl-1*cKO females and controls were used for histomorphometric ovarian analyses. Bax deficiency alone substantially increased the cohort of PMFs; and the *Mcl-1*c/*Bax*DKO ovarian reserve resembled *Bax*KO ovaries in all follicle numbers, implying a rescue of the *Mcl-1*-deficient oocyte phenotype (Figure 6a, Supplementary Figure S6A). Moreover, *Mcl-1*c/*Bax*DKO females displayed restored ovulation rates (Figure 6b, Supplementary Figure S6B), and a resiliency of MII oocytes against cellular fragmentation during *in vitro* culture, akin to wild-type controls and *Bax*KO (Figure 6c, Supplementary Figure S6C). However, despite a very robust rescue to the ovarian follicle pool, Bax deficiency was not able to restore mitochondrial functionality (Figure 6d, Supplementary Figure S6D) or correct the choromosomal misalignments (Figure 6e) in ovulated oocytes, indicating that, in oocytes, *Mcl-1* regulates additional aspects of cellular and mitochondrial physiology independent of Bax or cell death. Indeed, breeding trials of older (4- to 8-month old) *Bax*KO or *Mcl-1*c/*Bax*DKO mice revealed a longer breeding expectancy, but not an increase in the number of pups, as litter size remained <2. Although no *Mcl-1c*KO mice (*n*=8) produced litters after 4 months of age, two out of three *Mcl-1*c/*Bax*DKO females produced at least one litter during the 3-month breeding trial (Supplementary Table S1).

## Discussion

Limited ovarian phenotypes caused by deletion of anti-apoptotic Bcl-2 members^16, 17, 18, 19, 21^ has led to the postulation that they are either uninvolved or redundant in postnatal oocyte survival. Mcl-1 has a well-documented cell survival role for numerous somatic cell types;^36, 47, 48^ however, its necessity for long-term germ cell viability has not been established. We present evidence of Mcl-1 as the first pro-survival Bcl-2 member required for life-time maintenance of the ovarian reserve and stage-specific inhibition of varying modes of oocyte cell death.

As demonstrated above, cytoplasmic MCL-1 expression increases during the transition from PMF to PF, and continues to accumulate with sustained follicle growth; but oocytes in atretic follicles lack MCL-1 indicating that downregulation of MCL-1 could precede initiation of oocyte atresia. Mcl-1 appears to mediate PMF survival by antagonizing Bax action, as *Mcl-1* deficiency-induced PMF loss by apoptotic oocyte death, was restored by concurrent Bax ablation. Those *Mcl-1-*deficient oocytes that escape the early PMF demise and begin to grow, exhibit increased markers of cellular autophagy without apoptotic activation, resulting in only mildly elevated rates of atresia. This lack of apoptotic activation may in part be due to additional anti-apoptotic Bcl-2 members neutralizing activated BAX in the absence of MCL-1, or via additional undetermined mechanisms. We also show that the ability to initiate the apoptotic cascade does not occur till ovulation, as meiotic completion is essential for apoptotic activation.^49, 50^

We postulate that *Mcl-1*-deficient GV oocytes activate the autophagic machinery in response to mitochondrial dysfunction accompanied by disruption of metabolic machinery, however, we cannot exclude the possibility that Mcl-1 can directly regulate autophagy. Deletion of Mcl-1 has been recently linked to elevated mitochondrial dysfunction and activation of either autophagy or apoptosis in a stress- and cell-specific manner in cardiomyocytes, cortical neurons and MEFs.^28, 35, 36^ When evaluated, these phenotypes were accompanied by disruption of mitochondrial membrane potential, defective ATP output and production of superoxides. Mitochondrial bioenergetic output and functionality have long been considered essential factors in mediating oocyte quality and reproductive competence.^32, 33^ *Mcl-1*cKO oocytes display enhanced mitochondrial dysfunction; with elevated superoxides, impaired mitochondrial activity and reduced fumarate and malate, associated with chromosome abnormalities and elevated MII oocyte fragmentation; the last phenotype alone being mitigated by concurrent Bax deficiency. These mitochondrial phenotypes may be regulated by a newly identified matrix-localized isoform of MCL-1, associated with prevention of defective complex II enzymatic activity and disrupted supercomplex assembly.^35^ Confirmation of a role for this isoform in oocytes is yet to be determined. As oocyte-cumulus cell contact is required for the continuance of meiotic arrest, oocyte growth^51, 52^ and regulation of metabolite supply to the oocyte,^53, 54, 55, 56^ it is conceivable that maintained granulosa/cumulus cell support permits the *Mcl-1*-deficient immature oocytes to prolong their demise and, in conjunction with activation of autophagy, compensates for associated metabolic deficiencies. Subtle changes in ATP levels^57, 58^ and relief of MCL-1 inhibition of Beclin,^27, 28^ could be sufficient for autophagic activation in *Mcl-1*-deficient oocytes.

Additional studies are required to elucidate whether the maintenance of mitochondrial dysfunction with concomitant Bax deficiency may be due to a BAX-independent function of MCL-1, an additional role of BAX in mitochondrial function^59^ or some combination of both. As breeding was prolonged but not fully rescued by Bax deficiency, it indicates that although oocyte death could be prevented by Bax ablation, additional roles of Mcl-1, independent of Bax, are present, and may also govern embryo development.^60^ It would also be very informative to determine if depletion of Bok, another multi-channel pro-apoptotic member of Bcl-2 gene family, could rescue the developmental competence of *Mcl-1-*deficient oocytes. It should also be noted that in keeping with previous data,^46^ *Bax*-deficient females do display variable breeding performance.

Fertility and reproductive proficiency has been well established to rely on the maintenance of the ovarian reserve in addition to preservation of oocyte quality. We observed a correlation between maternal age and *MCL-1* expression levels in human oocytes, and our animal model demonstrates that oocyte-specific ablation of *Mcl-1* results in the accumulation of defects associated with compromised oocyte quality.^6, 7, 9, 10, 61^ Furthermore, we establish that Mcl-1 has the defining role in mediation of oocyte survival via protection of the postnatal PMF pool, in addition to the growing and ovulated oocyte pool. Thus, investigation into modulation of Mcl-1 expression by oocytes could prove informative in understanding what factors may contribute to POF and age-related oocyte loss.

## Materials and Methods

### Animals

*Mcl-1*^*tm3Sjk*^ (*Mcl-1*^*fl*^)^47^ mice carrying the floxed allele were obtained from the breeding colony of Dr. Korsmeyer and were intercrossed to mice carrying the Tg(Zp3-Cre)3Mrt (Zp3-Cre) transgene^62^ and to *Bax*^*tm1Sjk*^ mice.^63^ All mice were housed with free access to food and water and maintained on a 12- h:12- h light–dark cycle. All mouse experiments were performed in accordance with the Canadian Council on Animal Care (CCAC) guidelines for Use of Animals in Research and Laboratory Animal Care, under protocols approved by animal care committees of the Toronto Centre for Phenogenomics (TCP). To assess timing of excision, Zp3-Cre mice^62^ were also crossed to reporter lines Tg(CAG-Bgeo/ALPP)^1Lbe^ (Z/AP)^64^ and Gt(ROSA)26Sor^tm9(CAG-tdTomato)Hze^ (tdTomato).^65^

Animals were genotyped for possession of either *Mcl-1*^*+*^ or *Mcl-1*^*f*^ alleles using primers 5'-CTGAGAGTTGTACCGGACAA-3' (7MCL1) and 5'-GCAGTACAGGTTCAAGCCGATG-3' (6MCL1), and for *Mcl-1*^*null*^ (*Mcl-1*^*−*^) allele using primers 7MCL1 and 5'-ACGCTCTTTAAGTGTTTGGCC-3' (2MCL1). Presence of Zp3-Cre transgene was assessed using 5'-TGATGAGGTTCGCAAGAACC-3' (CREF) and 5'-CCATGAGTGAACGAACCTGG-3' (CRER) and genotyping for *Bax*^*+*^ *and Bax*^*−*^ alleles utilized 5'-GAGCTGATCAGAACCATCATG-3' (BAX-EX5-F), 5'-GTTGACCAGAGTGGCGTAGG-3' (BAX-LN5-R) and 5'-CCGCTTCCATTGCTCAGCGG-3' (BAX-NEO).

### Collection of oocytes and breeding

Immature (GV) oocytes were collected from antral follicles 46–48 h after pregnant mare's serum gonadotropin (PMSG; NHPP, Torrance, CA, USA or ProSpec, Rehovot, Israel (HOR-272)) priming and manually stripped of cumulus cells using narrow glass pipettes. For mature ovulated oocytes (MII), human chorionic gonadotropin (Sigma, Oakville, ON, Canada) priming was performed 44–48 h after PMSG and oocytes collected 14–16 h later; with cumulus removed by incubation in Hyaluronidase (Sigma). MII oocyte fragmentation rates were performed by MII culture in HTF media (Life Global, Guilford, CT, USA) supplemented with 0.1% BSA (Sigma) for 24 h. For breeding rates, dams at 4–5 weeks were mated to young wild-type males for a 6-month breeding trial and total litter number and size recorded. For *Mcl-1c/Bax*DKO or *Bax*KO breedings, dams at 4–8 months of age were bred to wild-type (*Mcl-1*^*f/+*^) males for 2–3 months and litters were recorded. Immature human oocytes, obtained from patients undergoing IVF treatment, were donated to research after obtaining patient consent approved by the Research Ethics Board at Mount Sinai Hospital, Toronto. Details of gene expression studies are described below.

### Real-time PCR with human oocytes

Obtained single human oocytes at GV stage free of cumulus cells were loaded into guanidine iso-thiocyanate solution and total nucleic acids were precipitated using glycogen as a carrier.^22^ Upon treatment with DNase (Sigma-Aldrich, St. Louis, MO, USA), reverse transcription was performed on the whole sample using Revert Aid Kit (Fermentas, Invitrogen Life Technologies, Burlington, ON, Canada) and oligo-dT primers. cDNA product was used in a real-time PCR reaction using LightCycler 480 SYBR Green I Master (Roche Applied Science, Indianapolis, IN, USA) and LightCycler 480 (Roche, Mannheim, Germany). The amplification profile included a pre-incubation step at 95 °C for 5 min, followed by denaturation at 95 °C, annealing at 62 °C and extension at 72 °C. The target gene concentration for the oocyte samples was extrapolated utilizing the standard curve for each target and the data were expressed as relative ratio to C_P_ value of *β-ACTIN*. Primer sequences for all genes analyzed (Supplementary Table S3).

### Histological ovarian analyses

Ovaries from *Mcl-1*^*f/−*^: Zp3-Cre (*Mcl-1*cKO), *Mcl-1*^*f/−*^, *Mcl-1*^*f/f*^, *Mcl-1*^*+/+*^, *Mcl-1*^*+/+*^: Zp3-Cre, *Mcl-1*^*f/+*^: Zp3-Cre, *Bax*^*−/−*^, *Mcl-1*^*f/+*^: *Bax*^*−/−*^: Zp3-Cre and *Mcl-1*^*f/−*^: *Bax*^*−/−*^: Zp3-Cre females were collected at varying timepoints (6 months, 3 months, 3 weeks, PN14 or PN7) and fixed in Dietrichs (4% formalin, 28% EtOH, 0.34N Glacial Acetic Acid (Sigma)) or 10% formalin (Fisher, Ottawa, ON, Canada) and following standard dehydration protocols were embedded in paraffin wax and sectioned (5 *μ*m) using a LEICA (Concord, ON, Canada) RM2255 Microtome and then mounted on Superfrost plus slides. Sections fixed in Dietrichs were rehydrated and stained with a picric acid/methyl blue stain, allowing for better resolution for histomorphometric analyses. Every third section was counted for PN7 and PN14 ovaries, every fifth section counted for 3 weeks ovaries, and every 10th section for 3 months and 6 months. Oocytes with visible nuclei from primordial, primary, secondary and antral follicles were quantitated and recorded and multiplied by associated factor (x3, x5 and x10) to gain an approximately full representation of the ovary. Atretic follicles were counted in sections from 3-week ovaries and are shown as a proportion of the total post-secondary growing follicles.

Ovaries from embryonic day 17.5 and PN3 tdTomato: Zp3-Cre animals were removed from animals and washed in mHTF. Entire tissue samples were viewed under LEICA DMI60003 Spinning Disc Confocal or LEICA MZ 165A Stereomicroscope using TRITC-Red laser (561 excitation, 620 emission), and imaged. Cryosections of ovaries from Z/AP: Zp3-Cre mice were postfixed in 0.2% glutaraldehyde and endogenous alkaline phosphatase (AP) activity was inactivated by heating for 30 min in PBS at 70 °C and then rinsed in PBS. Sections were then washed in buffer (100 mM Tris-HCL pH 9.5, 100 mM NaCl, 10 mM MgCl_2_) for 10 min and stained with NBT/BCIP stain, washed in PTM (0.1%Tween20, 2 mM MgCl_2_, in PBS) and counterstained with Nuclear Fast Red.

Sections fixed in 10% formalin were rehydrated and used for immunohistochemical staining protocols. Sections were submitted to antigen retrieval at ~95 °C for 10 min in sodium citrate buffer (10 mM tri-sodium citrate (Sigma) pH 6.0 with HCl), washed and blocked in 10% normal horse serum (NHS) for 1 h before overnight incubation in primary antibody (in 10% NHS) at 4 °C. Primary antibodies utilized include Mcl-1 (Rockland Immunochemicals, Limerick, PA, USA, 600-401-394S). Sections were then washed in PBS and incubated with secondary protocols from ABC Vectastain kit (PK-4001; Vector Labs, Burlington, ON, Canada) and then visualized using diamino-benzidine (DAB) (Sigma) substrate. After time-sensitive stain development, sections were counterstained in hematoxylin (Sigma) for identification of cell nuclei.

### TUNEL analyses

For detection of apoptotic cells, sections of PN1 ovaries were fixed in formalin, rehydrated and incubated in Proteinase K followed by permeabilization with 0.1% Triton-X. Slides were incubated in Reaction Mix (4 *μ*M biotin16-dUTP (Roche), 1.5 *μ*M dATP, 1X NEB4 buffer, 4 U/ul TdT enzyme (Roche) for 1.5 h at 37 °C and incorporated nucleotides were detected with streptavidin ABC Vectastain (Vector Labs) and visualized with DAB.

### Mitochondrial analyses – live cell stains

MII ovulated oocytes of *Mcl-1cKO* and controls were collected and subjected to a number of assays for determination of mitochondrial function. Total and respiring mitochondria were stained using Mitotracker fluorescent dyes (MitoTracker Green FM (M7154), MitoTracker Red 580 (M22425); Molecular Probes, Invitrogen LifeTechnologies) added to HTF in 100 nM concentration for 30 min, then washed in mHTF and imaged. For total cellular levels of ROS, oocytes were incubated in 10 *μ*M 2′, 7′-dichlorofluorescein diacetate (DCFDA; Molecular Probes) or 5 *μ*M MitoSox (Molecular Probes) in HTF for 15 min, washed in mHTF and imaged. The performance of fluorescent probes utilized for detection of ROS, and various mitochondrial markers was validated (Supplementary Figures S5D and E), using MII oocytes cultured in the presence of inducers of apoptosis or inhibitors of the electron transport chain. Induction of apoptosis in ovulated oocytes was done as previously described^66^ with 200 nM doxorubicin (DXR; Alexis, Enzo LifeSciences, Farmingdale, NY, USA) for 20 h followed by incubation with MitoTracker Green, MitoTracker Red, DCFDA or MitoSox. For disruption of mitochondrial function and inhibition of electron transport chain, MII oocytes were incubated in 100 nM complex III inhibitor Antimycin (Sigma) or vehicle (ethanol) for 15 min followed by co-incubation with mitochondrial dyes. Death rates for MII oocytes with antimycin treatment are included in Supplementary Figure S5E.

For mitochondrial membrane potential, MII oocytes were incubated with 5 *μ*g/ml DePsipher (DePsipher, Trevigen (6300-100-K, Gaithersburg, MD, USA)) in HTF for 90 min then washed and imaged. The mitochondrial fluorescent distribution of each oocyte was visually separated into pattern 1 – oocytes with strong central green fluorescence distribution and peripheral scattering of polarized mitochondria (red); or pattern 2 – oocytes with diffuse distribution of green fluorescence and little to no peripheral polarized mitochondria (red).

To assess metabolic profile citrate, malate and fumarate levels, MII oocytes were first frozen on glass slides by dipping in cold isopentane. Oocytes were then freeze–dried and processed for metabolic profile using protocol delineated in Chi *et al.*^67^ ATP content in single oocytes was measured by Cell Titer GLO assay (Promega, Madison, WI, USA).

### Immunofluorescence staining

GV or MII oocytes from *Mcl-1*cKO and control females were fixed in 10% formalin for 10 min and used for staining of markers of apoptosis and autophagy. Oocytes were first transferred to cooling ~95 °C sodium citrate buffer for antigen retrieval for 10 min using pulled-glass pipettes. Oocytes were moved to three washes in 0.1% Triton-X in 10 mM PBS and then blocked in 10% NHS in PBS. Following this step, oocytes were incubated in primary antibody overnight at 4 °C. Primary antibodies used include anti-Beclin-1 (Santa Cruz Biotechnologies, Dallas, TX, USA, sc-11427), anti-LC-3 (MBL, EMD Millipore, Billerica, MA, USA, PM046), anti-Lamp1 (1D4B, Developmental Studies Hybridoma Bank, Iowa City, IA, USA), anti-Lamp2 (ABL-93, Developmental Studies Hybridoma Bank), anti-Bax-NT (Upstate, EMD Millipore; 06-49), anti-BAX (Alexis, 210-003), anti-tubulin (Invitrogen LifeTechnologies, A11126), anti-actin (Santa Cruz Biotechnologies, sc-1616), anti-AIF (Santa Cruz Biotechnologies, sc-9416), anti-Mcl-1 (Santa Cruz Biotechnologies, sc-819) and anti-Mcl-1 (Rockland Immunochemicals, 600-401-394S). After primary antibody incubation, oocytes were transferred to 0.1%TX washes and incubated in host-specific secondary antibody conjugated with Alexa Fluor dyes (Invitrogen LifeTechnologies) and counterstained with blue fluorescent 4′,6-diamidino-2-phenylindole (DAPI, Sigma). Stained samples were mounted on slides in 50% glycerol for imaging. For negative controls, oocytes were exposed to nonspecific IgG or secondary antibody alone.

For evaluation of apoptotic induction, cytochrome c release, AIF release and pan-caspase activity assays were performed on GV and MII oocytes with protocols modified from Carboxyfluorescein Multi-Caspase Activity Kit (Biomol, Enzo LifeSciences) and cytochrome c release kit (InnoCyte – Calbiochem, EMD MIllipore). A portion of denuded GV and MII oocytes were permeabilized for 10 min with digitonin buffer. Both permeabilized and non-permeabilized oocytes were then fixed, and processed through the staining. For AIF release, AIF (Santa Cruz Biotechnologies, sc-9416) was utilized instead of cytochrome c antibody. For pan-caspase activity, GV and MII oocytes were incubated in FML-VAD-FMK stock dissolved in HTF medium for 2.5 h and washed and transferred to fixative as indicated in caspase activity kit. Oocytes were then washed and transferred to DAPI for 10–15 min, then mounted on Superfrost slides in 50% glycerol and imaged, as mentioned previously. The efficacy of these markers of apoptotic induction was verified (Supplementary Figure S5D), using MII oocytes cultured in the presence of 200 nM DXR^66^ for 14 h (Bax, cytochrome c) or 20 h (pan-caspase). MII oocytes incubated with DXR for 24 h display death rates of 80%.

### Imaging

Stained oocytes were imaged using LEICA DMI60003 Spinning Disc Confocal microscope with appropriate filters (objectives: 10X/0.40NA, 20X/0.70NA). Images were acquired and analyzed using Volocity software (PerkinElmer, Waltham, MA, USA) with Z-stack images taken at 0.354 *μ*m increments across 10 *μ*m sections to the either side of the midpoint of the oocyte, and average mean fluorescent intensity obtained from each sample was used for comparative purposes and expressed as random fluorescent units (RFUs). Colocalization data images were analyzed using Imaris (Bitplane, Zurich, Switzerland) software.

Histological ovarian sections were imaged with a LEICA/LEITX DMRXE microscope (objectives: 5X/0.12 NA, 10X/0.30 NA, 20X/0.50 NA, 40X/0.85 NA, 63X/0.75 NA, 100X/1.30 NA). Whole ovary images of TdTomato: Zp3-Cre mouse line and controls were acquired using the LEICA MZ16FA stereomicroscope (objectives: 4X/0.45 NA), with appropriate filters.

### Western blots

Two hundred GV oocytes from approximately 8–10 *Mcl-1*cKO and *Mcl-1*^*+/+*^ females were collected and lysed in RIPA buffer. Lysates were separated by SDS-PAGE and then transferred on to a PVDF membrane. Blots were incubated with anti-Mcl-1 (Rockland Immunochemicals, 600-401-394S) or anti-actin (Santa Cruz Biotechnologies, sc-1616). Membranes were washed and then incubated with specific HRP-conjugated donkey anti-rabbit (Santa Cruz Biotechnologies) or HRP-conjugated donkey anti-goat (Santa Cruz Biotechnologies) and detected using enhanced chemiluminescence (Thermo Scientific, Burlington, ON, Canada) SuperSignal West Femto Chemiluminescent Substrate and then imaged on the VersaDoc 5000MP Imaging System (Bio-Rad, Mississauga, ON, Canada).

### Statistical analysis

Data were analyzed using either one-way ANOVA with Holm–Sidak multiple comparisons test (Breeding performance, histomorphometric analyses, PCC, PCC-C); or using non-parametric Kruskal–Wallis one-way ANOVA on ranks, followed by Dunns *post-hoc* test for comparisons between groups, when normality failed or sample sizes were vastly different (ovulation rates, active Bax (GV), beclin, LC-3, MitoTracker green and red, active Bax (MII), fragmentation rates, MCC-A, MCC-B, MitoSox, ROS, cytochrome c, caspase activity, chromosomal misalignments). Statistical measures for two groups were performed using the unpaired *t*-test (histomorphometric analyses, atresia rates, metabolites), two-way ANOVA (DePsipher) or *χ*^2^ test (chromosome misalignments; Figure 6). Association maternal age and gene expression was done by Pearson correlation. All analysis was done using the Sigma Stat (Systat, San Jose, CA, USA) or PRISM software package (Graph Pad, San Diego, CA, USA). In all cases, differences were considered significant if value reached *P*<0.05.

## Abbreviations

AIF: apoptosis-inducing factor
Bax: Bcl-2-associated X protein
Bcl-2: B-cell lymphoma 2
BaxKO: Bax knockout
GV: germinal vesicle
LAMP: lysosome-associated membrane protein
MAP1LC3A: microtubule-associated protein 1 light chain
Mcl-1: myeloid cell leukemia-1
Mcl-1cKO: Mcl-1 conditional knockout
Mcl-1c/BaxDKO: Double knockout with conditional Mcl-1 and total Bax deletion
MII: metaphase II
PA: preantral
PCD: programmed cell death
PF: primary follicle
PMF: primordial follicle
PMSG: pregnant mare's serum gonadotropin
PN: postnatal day
POF: premature ovarian failure
ROS: reactive oxygen species
SF: secondary follicle
TUNEL: Terminal deoxynucleotidyl transferase dUTP nick end labeling
Z/AP: lacZ/alkaline phosphatase reporter
ZP3: zona pellucida 3
