# Supplementary material for: Mcl-1 is a key regulator of the ovarian reserve
Source: Cell Death Dis. 2015 May 7;6(5):e1755–. doi: 10.1038/cddis.2015.95 (PMC4669721; doi:10.1038/cddis.2015.95)
Supplement: Supplementary Table 1 [file cddis201595x1.doc]

**Table S1. 4month breeding trial of females >4mths (bred with wildtype males)**

| **Genotype** | **# of litters** | **average # of pups/litter** |
| --- | --- | --- |
| *Mcl-1^+/+^* | 3 | 6.33 |
| *Mcl-1^+/+^* | 3 | 5 |
| *Mcl-1^+/+^* | 4 | 7.75 |
| *Mcl-1^+/+^* | 4 | 7.5 |
| *Mcl-1^+/+^* | 3 | 6.67 |
| *Mcl-1c*KO | 0 | 0 |
| *Mcl-1c*KO | 0 | 0 |
| *Mcl-1c*KO | 0 | 0 |
| *Mcl-1c*KO | 0 | 0 |
| *Mcl-1c*KO | 0 | 0 |
| *Mcl-1c*KO | 0 | 0 |
| *Mcl-1c*KO | 0 | 0 |
| *Mcl-1c*KO | 0 | 0 |
| *Mcl-1c/Bax*DKO | 2 | 2.5* |
| *Mcl-1c/Bax*DKO | 1 | 4 |
| *Mcl-1c/Bax*DKO | 2 | 1.5 |
| *Bax*KO | 2 | 5 |
| *Bax*KO | 2 | 5 |
| *Bax*KO | 1 | 1 |
| *Bax*KO | 0 | 0 |
| *Mcl-1^f/-^*;*Bax*KO | 3 | 8.67 |
| *Mcl-1^f/-^*;*Bax*KO | 1 | 4 |
| *Mcl-1^f/-^*;*Bax*KO | 1 | 7 |
| *Mcl-1^f/-^*;*Bax*KO | 2 | 2 |
| *Mcl-1^f/-^*;*Bax*KO | 2 | 4.5 |
|  |  |  |

*3 Runts
