# Supplementary material for: Mcl-1 is a key regulator of the ovarian reserve
Source: Cell Death Dis. 2015 May 7;6(5):e1755–. doi: 10.1038/cddis.2015.95 (PMC4669721; doi:10.1038/cddis.2015.95)
Supplement: Supplementary Table 2 [file cddis201595x2.doc]

**Table S2. Clinical Data for *MCL-1* Transcript Expression**

| Patient ID | AGE | DIAGNOSIS | Stimulation Protocol | Outcome | Average *MCL-1* Ratio |
| --- | --- | --- | --- | --- | --- |
| 1 | 24 | PCO | LP | negative | 1.032161552 |
| 2 | 26.11 | MF | LP | viable singleton | 1.01494139 |
| 3 | 30 | Tubal | LP | negative | 1.009598505 |
| 4 | 30 | Unexplained | LP | negative | 0.996027002 |
| 5 | 31 | MF | A | negative | 1.084599058 |
| 6 | 32 | MF | LP | viable twins | 1.034939748 |
| 7 | 33.6 | Unexplained | A | negative | 0.979275627 |
| 8 | 34 | Tubal | A | negative | 1.017683217 |
| 9 | 34 | Endo | A | chemical pregnancy | 1.037350475 |
| 10 | 35 | Tubal/Endo | LP | negative | 0.97379141 |
| 11 | 34.2 | MF | LP | negative | 0.996208652 |
| 12 | 34.3 | Tubal | LP | viable twins | 1.037962452 |
| 13 | 34.3 | MF | LP | negative | 0.972962375 |
| 14 | 35 | Tubal | LP | negative | 1.027025026 |
| 15 | 35 | MF | LP | viable twins | 1.043292529 |
| 16 | 35 | Unexplained | A | chemical pregnancy | 1.061403221 |
| 17 | 35.6 | Tubal/MF | A | chemical pregnancy | 1.009370161 |
| 18 | 35 | Unexplained | LP | chemical pregnancy | 0.956379448 |
| 19 | 36 | Tubal | LP | negative | 1.026597204 |
| 20 | 37 | MF | LP | negative | 0.955915137 |
| 21 | 37 | MF | A | chemical pregnancy | 1.000648062 |
| 22 | 41 | AMA | A | early pregnancy loss | 0.959485836 |
| 23 | 41 | AMA/MF | A | negative | 0.986857439 |
| 24 | 42 | AMA/Tubal | A | negative | 0.971622578 |

Diagnosis: (Tubal) Tubal occlusion; (MF) Male factor; (Endo) Endometriosis, Unexplained, (PCO) Polycystic Ovarian syndrome, (AMA) Advanced Maternal Age

Stimulation protocol: (LP) Long protocol or (A) Antagonist
