# Supplementary material for: Mcl-1 is a key regulator of the ovarian reserve
Source: Cell Death Dis. 2015 May 7;6(5):e1755–. doi: 10.1038/cddis.2015.95 (PMC4669721; doi:10.1038/cddis.2015.95)
Supplement: Supplementary Table 3 [file cddis201595x3.doc]

**Table S3. Primer sequences for Real Time PCR for human oocytes.**

| **Gene** | **Forward** | **Reverse** |
| --- | --- | --- |
| *BCL-2* | GGCCCTCCAGATAGCTCATT | CAAAAGCAAACCTTGGTTGAA |
| *BCL-2L1* | GAGCTGTTTATGGCCTCAGC | GCTCCCATAGCTGTTCCTGA |
| *BCL-2L10* | TGCCCAACTGTGACCAACTA | CCAAATCACCACCTCAGGAC |
| *BCL-2L11* | CACTGGACTGGGTGTCAAGA | CCACGTGTACGTGAGTGGAC |
| *MCL-1* | GGGAAAAACATGCAGTCCTC | TCCTGGCACAGCTATCAAAA |
| *β-ACTIN* | CACAGGGGAGGTGATAGCAT | CACGAAGGCTCATCATTCAA |
