# Supplementary material for: Mcl-1 is a key regulator of the ovarian reserve
Source: Cell Death Dis. 2015 May 7;6(5):e1755–. doi: 10.1038/cddis.2015.95 (PMC4669721; doi:10.1038/cddis.2015.95)
Supplement: Supplementary Figure Legends [file cddis201595x4.doc]

**Fig.S1. Confirmation of Cre excision.**

**(A)** Confirmation of oocyte-specific Zp3-Cre excision using tdTomato Reporter line. Cre mediated excision results in removal of *loxP*-flanked STOP cassette upstream of red fluorescent protein variant (tdTomato) marking activity of Cre in oocytes (arrows) (i) Ovaries from embryonic day 17 (E17) females of the Tomato Reporter line with and without Cre transgene and respective brightfield images below. (ii) Ovaries from PN3 Tomato Reporter line with and without Cre transgene with respective brightfield images below. **(B)** Adult ovaries of Z/AP Reporter line with Zp3-Cre transgene stained for alkaline phosphatase activity (blue) counterstained with eosin (pink). Excision is detected in oocytes within primordial follicles indicated by arrows **(C)** MCL-1 expression in *Mcl-1^f/-^*: Zp3-Cre (*Mcl-1*cKO) oocytes and controls utilizing immunohistochemistry of 3 week *Mcl-1*cKO and *Mcl-1^+/+^*: Zp3-Cre ovary. Lack of immunoreactivity for MCL-1 in growing oocytes in *Mcl-1*cKO (arrowheads) and robust expression in control (wildtype Zp3-Cre) ovaries (arrows) observed, with diminished MCL-1 levels in oocytes of atretic follicles (red arrowhead). **(D)** Western Blots (WB) of MCL-1 protein in 200 GV oocytes obtained from *Mcl-1*cKO and wildtype control with associated levels of internal control ACTIN.

**Fig.S2. Breeding performance, ovulation rates and histomorphometric analyses of ovaries. (A)** Cumulative pup number from breeding of *Mcl-1*cKO (n=6) in comparison to females of the additional control genotypes *Mcl-1^+/+^*: Zp3-Cre (n=4) and *Mcl-1^f/f^* (n=6). Females were mated with wildtype males over 6 month breeding trial. Each column represents individual females with each varied color segment indicative of individual litters with significant reduction in litter size and litter number in *Mcl-1*cKO females. *Mcl-1*cKO (n=7) females revealed significantly reduced average litter size compared to *Mcl-1^+/+^* (n=13), *Mcl-1^+/+^*: Zp3-Cre (n=6) and *Mcl-1^f/f^* (n=13) control females. Values represent average number of pups/litter ± SEM. **(B)** Ovulation rates from hormonally primed 6 month *Mcl-1*cKO (n=12), compared to *Mcl-1^+/+^*: Zp3-Cre (n=12), *Mcl-1^f/f^* (n=7), and *Mcl-1^f/-^* (n=8) control females. Ovulation rates from hormonally primed 3 month *Mcl-1*cKO (n=20), compared to *Mcl-1^+/+^*: Zp3-Cre (n=14), *Mcl-1^f/f^* (n=9), *Mcl-1^f/-^* (n=12), and *Mcl-1^f/+^*: Zp3-Cre (n=16) control females. Values in graph represent average oocytes ovulated per female +/- SEM. **(C)** Histomorphometric analyses of primordial, primary, secondary and preantral follicles in *Mcl-1*cKO (n=5), compared to *Mcl-1^+/+^*: Zp3-Cre (n=3), *Mcl-1^f/f^* (n=5), *Mcl-1^f/-^* (n=4), and *Mcl-1^f/+^*: Zp3-Cre (n=3) control females. Values represent average number of follicles/ovary ± SEM **(D)** Appearance of largest diameter histological section of *Mcl-1*cKO and *Mcl-1^+/+^*: Zp3-Cre ovaries at 3 months of age. Ovarian sections stained with nuclear hematoxylin. **(E)** Ovulation rates from primed 3 week *Mcl-1*cKO (n=21), compared to *Mcl-1^+/+^* (n=19) control females. Values represent average oocytes ovulated per female ± SEM. **(F)** Death rates based on TUNEL stain of PN1 ovaries of *Mcl-1*cKO (n=4) compared to *Mcl-1^+/+^* (n=3), *Mcl-1^f/-^* (n=4), and *Mcl-1^f/+^*: Zp3-Cre (n=4) control females counterstained with methyl green. Values represent average percentage of TUNEL positive/total PMF per section ± SEM (*= p<0.05, **= p<0.01, ***= p<0.001). Genotypes in legend apply to all graphs.

**Fig.S3. Markers of autophagy and apoptosis in GV oocytes. (A)** Isolated *Mcl-1*cKO (n=12), *Mcl-1^+/+^* (n=13) and *Mcl-1^f/+^*: Zp3-Cre (n=7) GV oocytes were stained for markers of activation of the apoptotic cascade. Representative images are of GV oocytes stained with anti-Bax NT (green) and counterstained with DAPI. Respective brightfield images of each genotype are displayed below, in addition to secondary antibody-only control images. The mean intensity of signal was quantitated per oocyte as the average fold change of RFUs per oocyte ± SEM normalized to average *Mcl-1^+/+^* value. **(B)** For the Cytochrome c release assay, *Mcl-1*cKO (n=19), *Mcl-1^+/+^* (n=33) and *Mcl-1^f/-^* (n=5) GV oocytes, and permeabilized *Mcl-1*cKO (n=13), *Mcl-1^+/+^* (n=31) and *Mcl-1^f/-^* (n=6) oocytes, were stained with anti-Cytochrome c (green) to determine the proportion of Cytochrome c retained in mitochondria. Mean signal intensity was quantitated and values represent the average fold change of RFUs per oocyte ± SEM of retained/total Cytochrome c, normalized to average *Mcl-1^+/+^* value. Differences were not significant. **(C)** For the AIF release assay, *Mcl-1*cKO (n=11), *Mcl-1^+/+^* (n=12) and *Mcl-1^f/-^* (n=8) GV oocytes, and permeabilized *Mcl-1*cKO (n=9), *Mcl-1^+/+^* (n=12) and *Mcl-1^f/-^* (n=6) oocytes, were stained with anti-AIF (green) to determine the proportion of AIF retained in mitochondria. Mean signal intensity was quantitated and values represent the average fold change of RFUs per oocyte ± SEM of retained/total AIF, normalized to average *Mcl-1^+/+^* value. Differences were not significant **(D)** *Mcl-1*cKO (n=12) and *Mcl-1^+/+^* (n=7) GV oocytes were assessed for pan-Caspase activity. Mean intensity was quantitated per oocyte and values indicate average RFUs per oocyte ± SEM. Differences were not significant. **(E)** Immunofluorescent stain of LC-3 (red) in *Mcl-1*cKO (n=23), *Mcl-1^+/+^* (n=23) and *Mcl-1^f/+^*: Zp3-Cre (n=12) isolated 3 week GV oocytes, counterstained with DAPI. Mean intensity signal of each oocyte was quantitated and values shown represent the average relative fluorescence units (RFU x10^4^) per oocyte ± SEM. **(F)** Confocal single layer images of Beclin-1 and LAMP-2 foci in isolated GV oocytes from 3 week ovaries, counterstained with nuclear fluorescent stain DAPI, with corresponding brightfield images and secondary antibody-only controls displayed below. Number and volume of Beclin-1 punctae were quantitated and values graphed represent average number and volume of Beclin-1 foci ± SEM, respectively. Coefficients of co-localization (Pearsons in region of interest (PCC), Pearsons in colocalized pixels (PCC-C), Manders A (MCC-A) and Manders B (MCC-B) were calculated for Beclin-1 (red) and LAMP2 (green), on n=19 *Mcl-1*cKO, n=13 *Mcl-1^+/+^* and n=10 *Mcl-1^f/+^*: Zp3-Cre GV oocytes and displayed in graphs (lower right). (*= p<0.05, **= p<0.01, ***= p<0.001). Genotypes in legend apply to all graphs.

**Fig.S4. Markers of mitochondrial function and chromosome alignment in ovulated oocytes. (A)** *Mcl-1*cKO (n=57), *Mcl-1^+/+^* (n=85), *Mcl-1^+/+^*: Zp3-Cre (n=54) and *Mcl-1^f/+^*: Zp3-Cre (n=102) MII oocytes were stained with a potential dependent mitochondrial dye (DePsipher). Values in graph (left) represent a proportion (%) of oocytes with few (<20) or numerous (>20) polarized (red) mitochondrial foci per *Mcl-1*cKO or *Mcl-1^+/+^* oocytes. Values in graph (right) represent the mitochondrial distribution of each oocyte separated into Pattern 1 or Pattern 2. **(B)** MII oocytes were stained with MitoTracker Green, which stains the total mitochondrial population of the cell. Representative images from n=41 *Mcl-1*cKO MII oocytes, and n=43 *Mcl-1^+/+^* MII oocytes are displayed, with respective brightfield images below. The mean intensity signal from these and n=25 *Mcl-1^f/+^*: Zp3-Cre MII oocytes were quantitated (right) and the values graphed represent average fold change ± SEM of quantitated relative fluorescence per oocyte, normalized to average *Mcl-1^+/+^* oocyte value. **(C)** *Mcl-1*cKO (n=68), *Mcl-1^+/+^* (n=52) and *Mcl-1^f/+^*: Zp3-Cre (n=10) MII oocytes were stained with MitoTracker Red, an indicator of actively respiring mitochondria. The mean intensity signal from these MII oocytes were quantitated and the values graphed represent average fold change ± SEM of quantitated relative fluorescence units (RFUs) per oocyte, normalized to wildtype oocytes. **(D)** *Mcl-1*cKO (n=15) and *Mcl-1^+/+^* (n=15) oocytes were assayed for metabolite levels of ATP and citrate. Values are represented by average metabolite level (µmol) per oocyte wet weight (kg) ± SEM. **(E)** MII oocytes were stained with MitoSox (red) (*Mcl-1*cKO (n=20) and *Mcl-1^+/+^* (n=34), *Mcl-1^+/+^*: Zp3-Cre (n=20) and *Mcl-1^f/+^*: Zp3-Cre (n=8)) or **(F)** DCFDA (green) (*Mcl-1*cKO (n=20), *Mcl-1^+/+^* (n=38) *Mcl-1^+/+^*: Zp3-Cre (n=20)) or. Values represent average fold change ± SEM of RFUs per oocyte, normalized to wildtype. **(G)** Additional examples of MII oocytes (*Mcl-1*cKO (n=84) and *Mcl-1^+/+^* (n=89), in addition to *Mcl-1^f/+^*: Zp3-Cre (n=160) and *Mcl-1^f/-^* (n=34) MII oocytes) stained with DAPI(red) and anti-TUBULIN (green) to visualize chromatin and spindle, with misaligned chromosomes indicated with white arrow**.** Values represent percentage of oocytes with misaligned chromosomes/total oocyte pool. (*= p<0.05, **= p<0.01, ***= p<0.001). Genotypes in legend apply to all graphs.

**Fig.S5. Markers of apoptosis in ovulated oocytes and positive controls. (A)** *Mcl-1*cKO (n=12), *Mcl-1^+/+^* (n=32), *Mcl-1^+/+^*: Zp3-Cre (n=18) and *Mcl-1^f/-^* (n=6) MII oocytes were stained for markers of activation of the apoptotic cascade. Displayed are representative images of MII oocytes stained with anti-Bax NT (green), indicative of potential for oligomerization via an N-terminal conformational change of BAX, and counterstained with DAPI. Respective brightfield images of each genotype and secondary antibody-only controls are displayed below. The mean signal intensity of each oocyte and n=7 *Mcl-1^f/-^* MII oocytes was quantitated and values graphed represent average relative fluorescence units (RFUx10^3^) ± SEM. Total BAX levels (below) displayed no change in quantitated *Mcl-1*cKO (n=12) and *Mcl-1^+/+^* (n=19) MII oocytes where values graphed represent average relative fluorescence units (RFUx10^3^) ± SEM. **(B)** *Mcl-1*cKO (n=19), *Mcl-1^+/+^* (n=33) and *Mcl-1^+/+^*: Zp3-Cre (n=12) MII oocytes were stained for total, and *Mcl-1*cKO (n=13), *Mcl-1^+/+^* (n=31) and *Mcl-1^+/+^*: Zp3-Cre (n=23) oocytes were stained for retained Cytochrome c (green). Values represent the average fold change of RFUs per oocyte ± SEM of retained/total Cytochrome c, normalized to average *Mcl-1^+/+^* value. **(C)** *Mcl-1*cKO (n=16), *Mcl-1^+/+^* (n=13) and *Mcl-1^f/+^*: Zp3-Cre (n=11) MII oocytes were stained for pan-Caspase activity (green). Values represent the average fold change of RFUs per oocyte ± SEM normalized to average *Mcl-1^+/+^* value **(D)** Positive Control Experiments. MII Oocytes treated with doxorubicin (200nM) for 14hrs (anti-BaxNT, Cytochrome c retention) and 20hrs (pan-caspase activity, mitochondrial markers). DXR treated MII oocytes displayed increased Bax-NT (n=15), reduced cytochrome c (n=11) mitochondrial retention and increased caspase activity (n=25), compared to untreated controls (n=13, n=10, n=12, respectively). Values graphed represent average relative fluorescence units ± SEM. DXR treated MII oocytes also displayed reduced MitoTracker Red (n=31), unchanged MitoTracker Green (n=31) and elevated MitoSox (n=24) and ROS (n=29), compared to untreated controls (n=27, n=27, n=19, n=15, respectively). Values represent the average fold change of RFUs per oocyte ± SEM, normalized to average *Mcl-1^+/+^* value. **(E)** Positive Control Experiments. MII oocytes were treated with Antimycin (50nM or 100nM) for 6hrs to demonstrate elevated death rates compared to controls. MII oocytes treated for 15min with 100nM Antimycin display reduced MitoTracker Red (n=19), MitoTracker Green (n=19), MitoSox (n=18) and ROS (n=18) levels compared to untreated controls (n=19, n=19, n=19, n=19, respectively); consistent with inhibition of electron transport chain. Values represent the average fold change of RFUs per oocyte ± SEM, normalized to average *Mcl-1^+/+^* value. *= p<0.05, **= p<0.01, ***= p<0.001). Genotypes in legend apply to all graphs.

**Fig.S6. Rescue of *Mcl-1*-deficient follicle loss by concurrent *Bax*-ablation. (A)** Histomorphometric analyses of 3 month ovaries in *Mcl-1*cKO (n=5), *Mcl-1^+/+^* (n=3), *Mcl-1^f/+^*: Zp3-Cre (n=3), *Mcl-1^f/+^*:Zp3-Cre:*Bax*KO (n=3) and *Mcl-1*c/*Bax*DKO (n=3) females. Values represent average follicle number in ovary per genotype ± SEM. **(B)** Ovulation rates of *Mcl-1*cKO (n=20), *Mcl-1^+/+^* (n=12), *Mcl-1^f/+^*: Zp3-Cre (n=16), *Mcl-1^f/+^*:Zp3-Cre:*Bax*KO (n=7) and *Mcl-1*c/*Bax*DKO (n=3) 3 month females. Values represent average number of ovulated oocytes ± SEM. **(C)** MII oocytes from *Mcl-1*cKO (n=6), *Mcl-1^+/+^* (n=10), *Mcl-1^f/+^*: Zp3-Cre (n=4), *Mcl-1^f/+^*:Zp3-Cre:*Bax*KO (n=3) and *Mcl-1*c/*Bax*DKO (n=3) females (n=104, n=180, n=69, n=54 and n=48 oocytes, respectively) cultured for 24hr and values represent the average percentage of fragmented oocytes per female ± SEM. **(D)** *Mcl-1*cKO (n=50), *Mcl-1^+/+^* (n=33), *Mcl-1^f/+^*:Zp3-Cre:*Bax*KO (n=7) and *Mcl-1*c/*Bax*DKO (n=49) MII oocytes were stained with MitoTracker Red. Values represent fold change of average RFUs per oocyte ± SEM normalized to average *Mcl-1^+/+^* value. *Mcl-1^f/+^*:Zp3-Cre:*Bax*KO and *Mcl-1*c/*Bax*DKO data were all graphed against *Mcl-1*cKO and *Mcl-1^+/ +^* data from figures S2, S4 and 5. (*= p<0.05, **= p<0.01, ***= p<0.001). Genotypes in legend apply to all graphs.
