# Supplementary figures and images for: Mcl-1 is a key regulator of the ovarian reserve
Source: Cell Death Dis. 2015 May 7;6(5):e1755–. doi: 10.1038/cddis.2015.95 (PMC4669721; doi:10.1038/cddis.2015.95)

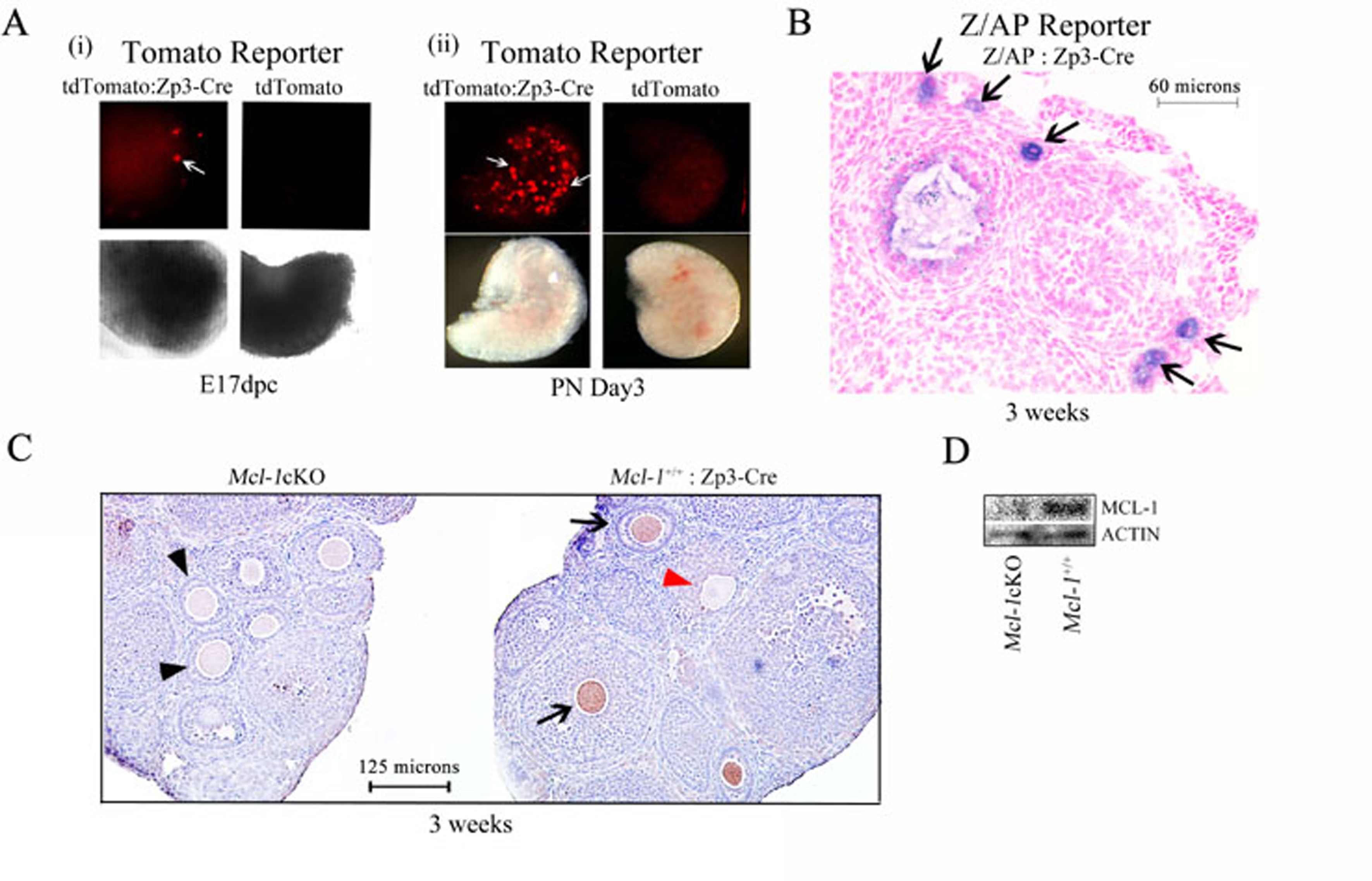

Supplement: Supplementary Figure 1 [file cddis201595x5.tif]

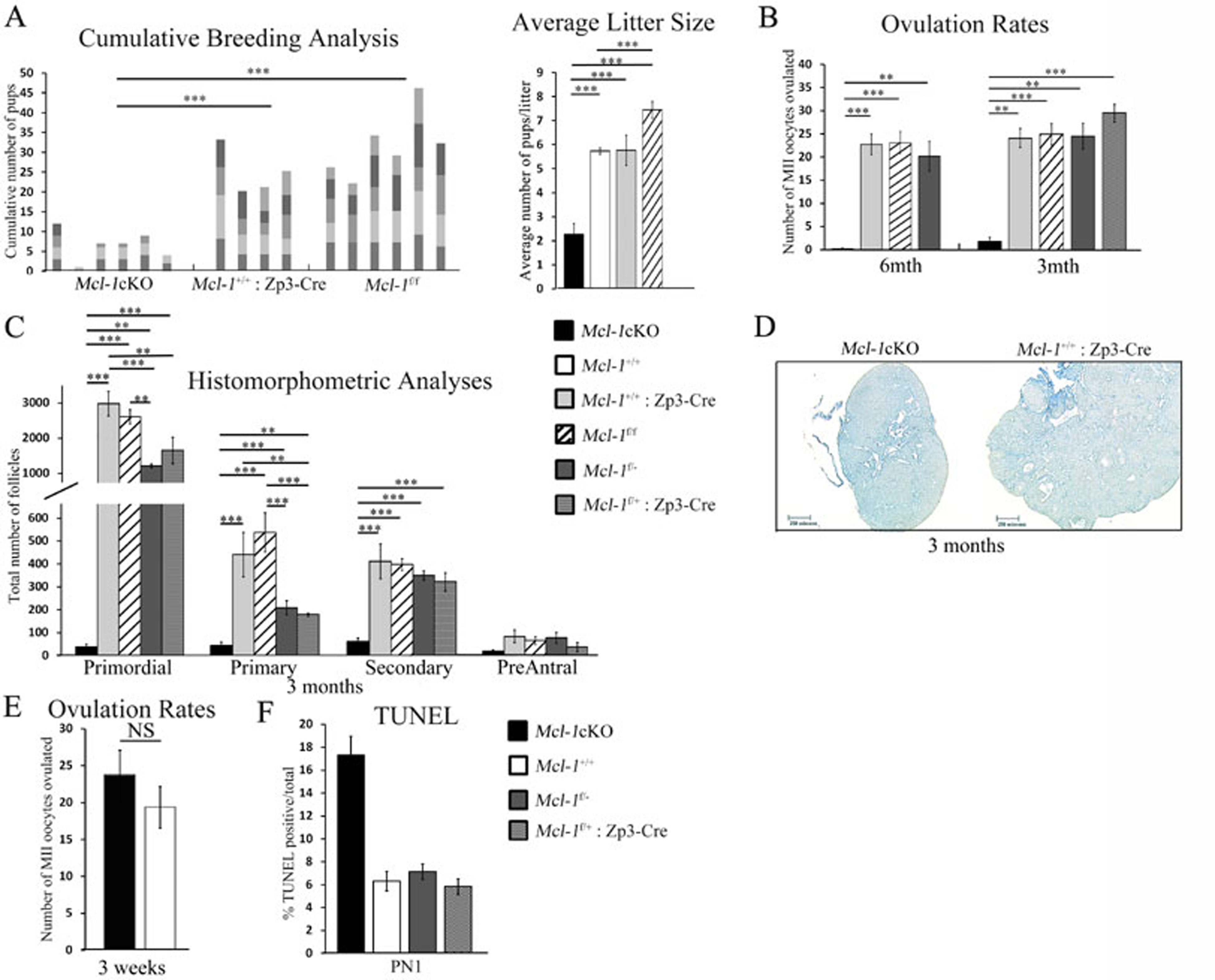

Supplement: Supplementary Figure 2 [file cddis201595x6.tif]

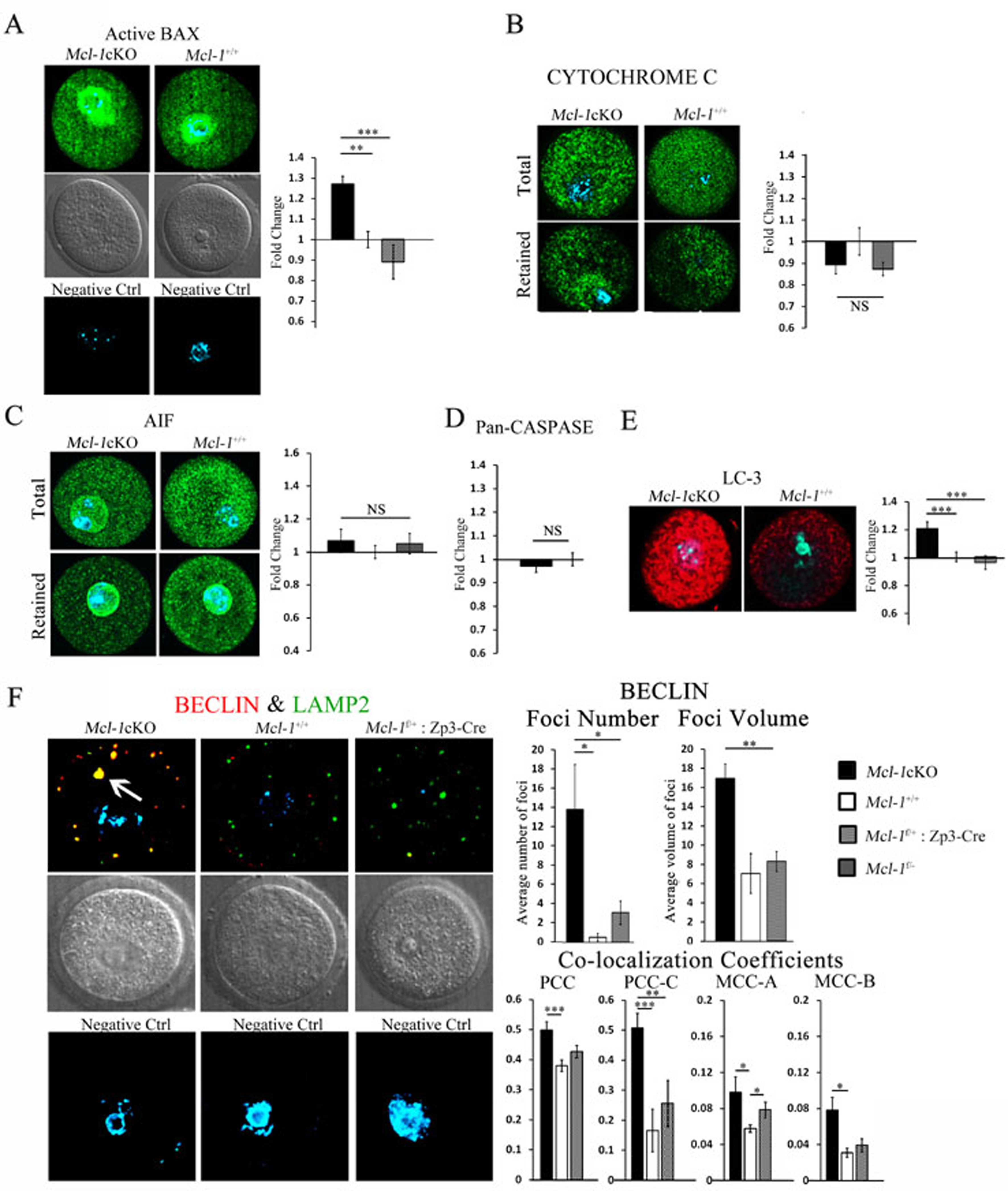

Supplement: Supplementary Figure 3 [file cddis201595x7.tif]

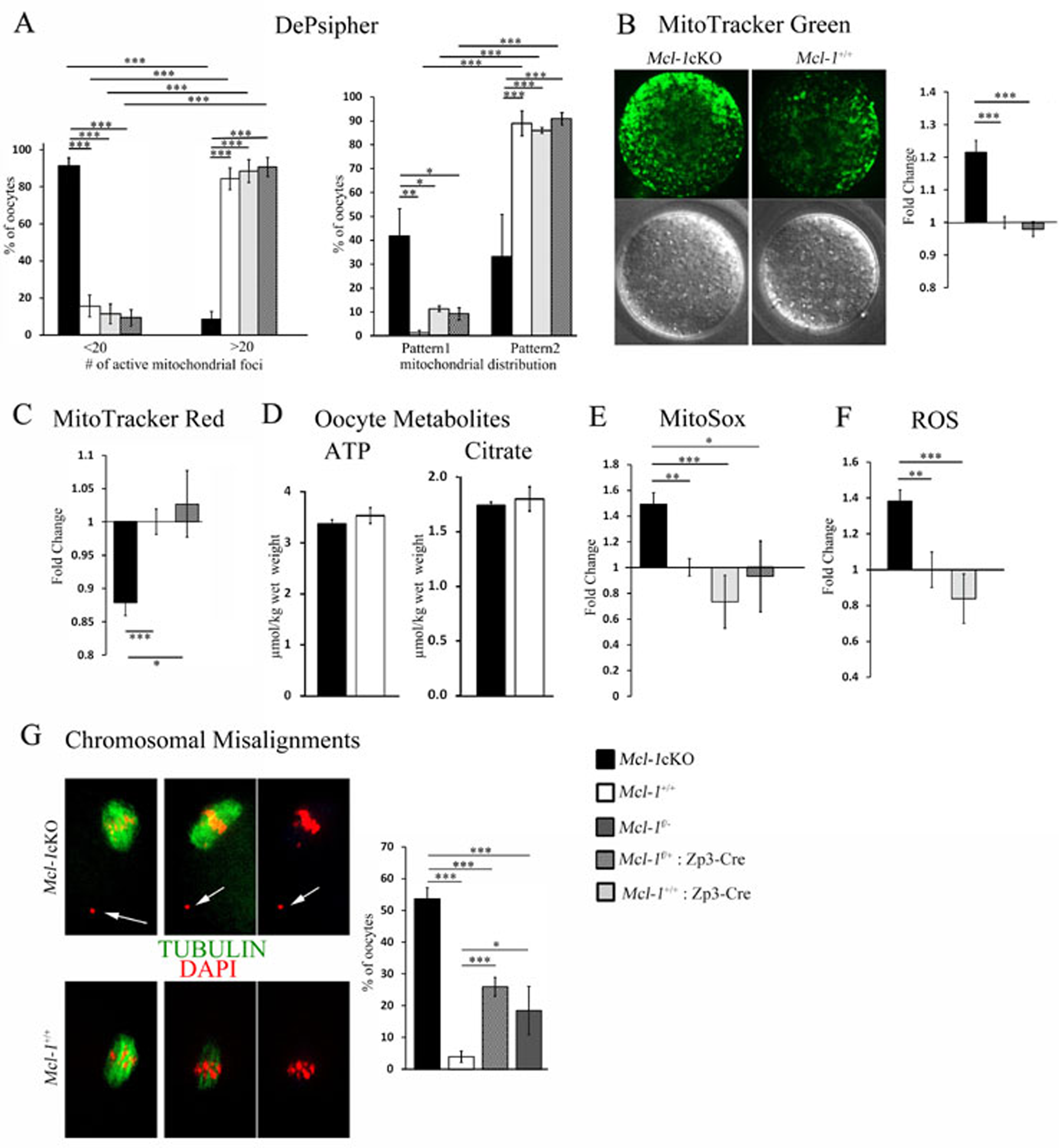

Supplement: Supplementary Figure 4 [file cddis201595x8.tif]

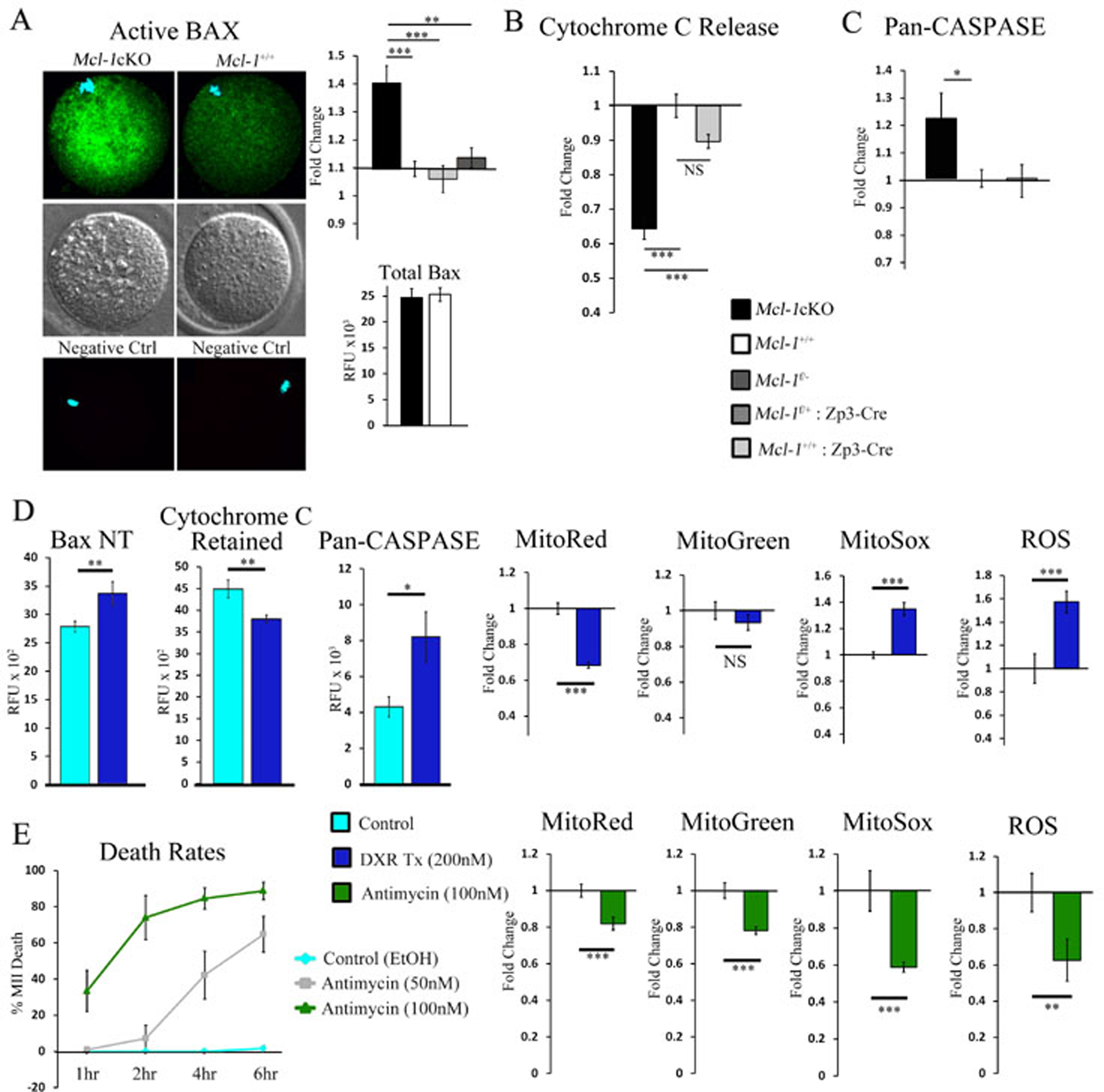

Supplement: Supplementary Figure 5 [file cddis201595x9.tif]

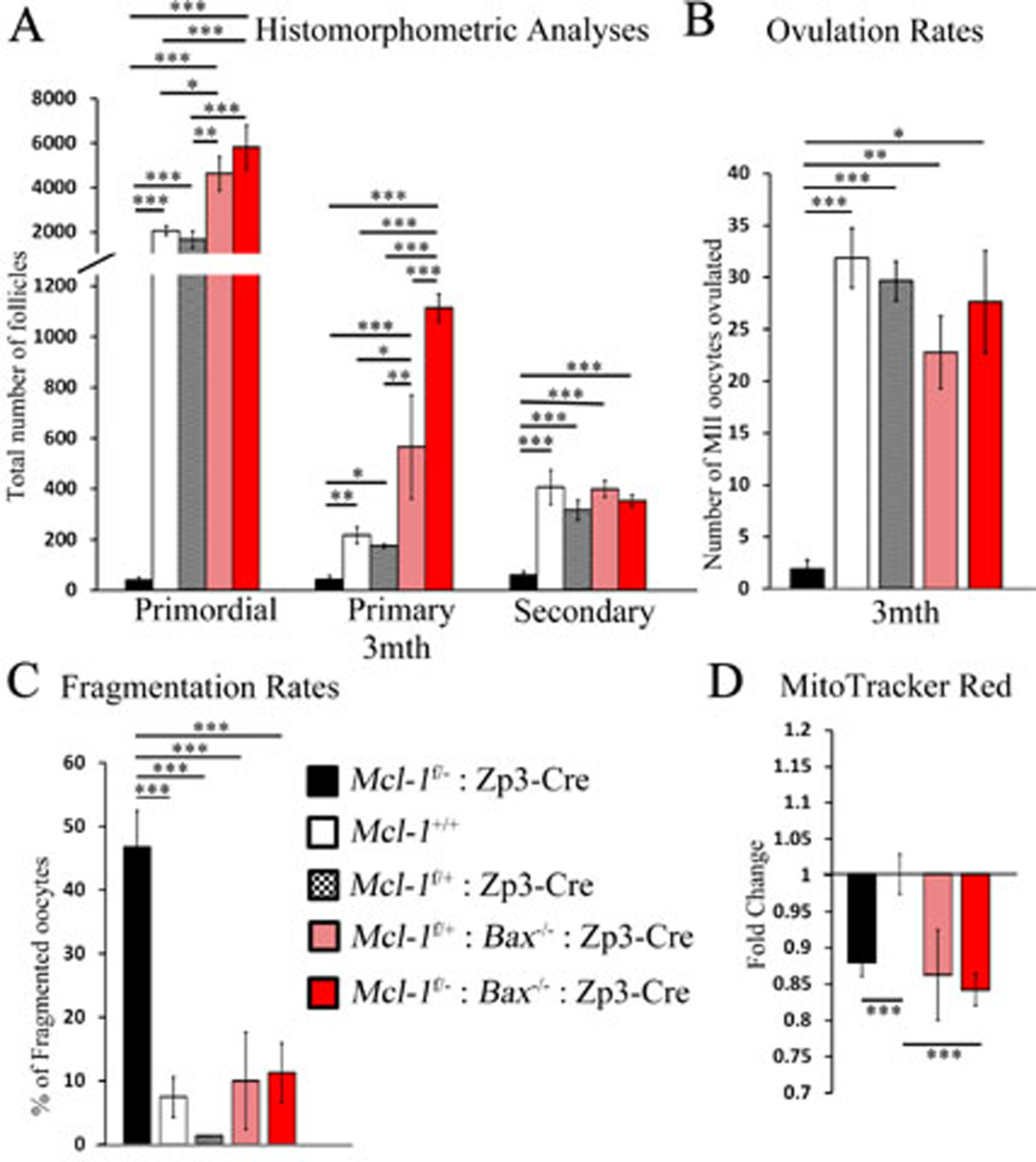

Supplement: Supplementary Figure 6 [file cddis201595x10.tif]
